# Dissecting transcription of the 8q24-MYC locus in prostate cancer recognizes the equilibration between androgen receptor direct and indirect dual-functions

**DOI:** 10.1186/s12967-023-04429-4

**Published:** 2023-10-12

**Authors:** Ju Guo, Zhao Wei, Tianwei Jia, Liyang Wang, Nuosu Nama, Jiaqian Liang, Xinghua Liao, Xiaming Liu, Yanfei Gao, Xiaoqiang Liu, Keshan Wang, Bin Fu, Shaoyong Shawn Chen

**Affiliations:** 1 Department of Urology, The First Affiliated Hospital of Nanchang University, Yongwai Street 17, Nanchang, 330006 China; 2 Department of Clinical Laboratory, Qilu Hospital of Shandong University, Jinan, 250012 Shandong China; 3 Department of Clinical Laboratory, The Second Hospital, Cheeloo College of Medicine, Shandong University, Jinan, 250033 Shandong China; 4 Shandong Engineering & Technology Research Center for Tumor Marker Detection, Jinan, 250033 Shandong China; 5 Shandong Provincial Clinical Medicine Research Center for Clinical Laboratory, Jinan, 250033 Shandong China; 6 Hematology-Oncology Division, Department of Medicine, Beth Israel Deaconess Medical Center and Harvard Medical School, Boston, MA 02215 USA; 7 Department of Cell Development Biology, College of Life Sciences, Shaanxi Normal University, Xi’an, 710119 ShanXi China; 8 Department of Epidemiology, Johns Hopkins Bloomberg School of Public Health, 615 North Wolfe Street, Baltimore, MD 21205 USA; 9 Department of Urology, Wuhan No. 1 Hospital, No. 215 Zhongshan Avenue, Wuhan, China; 10 Institute of Biology and Medicine, College of Life and Health Sciences, Wuhan University of Science and Technology, No. 947, Heping Avenue, Qingshan District, WuHan, 430081 Hubei China; 11 Department of Urology, Tongji Hospital, Tongji Medical College, Huazhong University of Science and Technology, 1095 Jiefang Avenue, Wuhan, 430030 Hubei China; 12 Center for Medical Epigenetics, School of Basic Medical Sciences, Chongqing Medical University, 1 Yixueyuan Road, Chongqing, 400016 People’s Republic of China; 13 Department of Urology, Union Hospital, Tongji Medical College, Huazhong University of Science and Technology, 1277 Jiefang Avenue, Wuhan, 430022 China; 14 Institute of Urology, Union Hospital, Tongji Medical College, Huazhong University of Science and Technology, 1277 Jiefang Avenue, Wuhan, 430022 China

**Keywords:** Androgen receptor, Transcriptional dual-functions, 8q24-MYC locus, AR binding sites, CRIPSR/Cas9 genomic knock-out, Co-factor redistribution, Prostate cancer

## Abstract

**Background:**

Androgen receptor (AR) activation and repression dual-functionality only became known recently and still remains intriguing in prostate cancer (PCa). MYC is a prominent oncogene that functionally entangles with AR signaling in PCa. Further exploration of AR regulatory mechanisms on MYC gene transcription bears clinical and translation significance.

**Methods:**

Bioinformatics analysis of PCa cell line and clinical RNA-Seq and ChIP-Seq (chromatin immunoprecipitation-sequencing) datasets to anchor interactions of AR and MYC transcriptional networks. ChIP-qPCR and 3C (chromosome conformation capture) analyses to probe MYC distal regulation by AR binding sites (ABSs). CRISPR/Cas9-mediated genome-editing to specify functions of ABS within the 8q24-MYC locus on androgen-mediated MYC transcription. Global FoxA1 and HoxB13 distribution profiling to advance AR transcriptional mechanisms.

**Results:**

Here we recognize AR bi-directional transcription mechanisms by exploiting the prominent 8q24-MYC locus conferring androgen hyper-sensitivity. At ~ 25 Kb downstream of the MYC gene, we identified an undefined ABS, P10. By chromatin analyses, we validated androgen-dependent spatial interaction between P10 and MYC-Promoter (MYC-Pro) and temporal epigenetic repression of these MYC-proximal elements. We next designed a CRISPR/Cas9-mediated double genomic knock-out (KO) strategy to show that P10-KO slightly lessened androgen-elicited MYC transrepression in LNCaP-AR cells. In similar genomic editing assays, androgen-mediated MYC repression became slightly deepened upon KO of P11, an ABS in the PVT1 gene locus highly enriched in AR-binding motifs and peaks. We also investigated multiple ABSs in the established PCAT1 super-enhancer that distally interacts with MYC-Pro for transactivation, with each KO pool consistently shown to relieve androgen-elicited MYC repression. In the end, we systemically assessed androgen effects in the 8q24-MYC locus and along PCa genome to generalize H3K27ac and BRD4 re-distribution from pioneer factors (FoxA1 and HoxB13) to AR sites.

**Conclusion:**

Together, we reconciled these observations by unifying AR dual-functions that are mechanistically coupled to and equilibrated by co-factor redistribution.

**Graphical Abstract:**

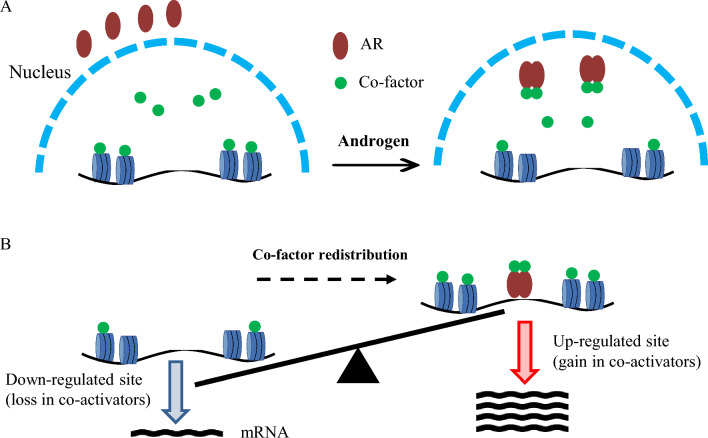

**Supplementary Information:**

The online version contains supplementary material available at 10.1186/s12967-023-04429-4.

## Introduction

The advancements in chromatin and epigenetics technologies in recent years have enabled the exposure of AR transrepressive functions that remain elusive for decades [1–3]. In contrast to its classic transactivator role that roots in specific chromatin occupy, AR-mediated transrepression is in dissociation with DNA-binding [2, 3]. In conjunction with AR direct and indirect dual-functions, AR signature genes exhibited distinct profiles in terms of timing and magnitudes of androgen responsiveness [2–4]. These observations argue against previously oversimplified views on AR functionalities, pointing towards an extrapolation that AR-dependent transcription is in dynamic equilibration between androgen direct and indirect actions.

MYC is a prominent oncogene functionally entangled with AR signaling and its overexpression renders androgen insensitivity and invasive PCa transformation [5–9]. Clustered in the 8q24 TAD (topologically associating domain), MYC and flanking lncRNAs are components of integrated oncogenic networks [10–13]. It is speculated that the mystic gain in MYC regulation accounts for AR transition from a tumor-suppressor to an oncogene [14]. However, AR regulatory mechanisms on MYC gene transcription have been controversial for decades: the effects are tissue-specific and cell-specific and can be either stimulatory or repressive. In addition, AR and MYC transcriptional networks are mutually repressive and functionally compensatory, mediated by competition for shared co-factors [3, 15, 16]. Considering AR and MYC each regulates a transcriptional network covering hundreds of genes, we conceive the spectral exchanges between their transcriptomes would cause massive co-factor exchanges.

MYC could trigger immediate and reversible cancer-driven signals, making it compelling to specify the regulatory mechanisms of the MYC gene [17]. MYC is also known for its vulnerability in mRNA and protein stability and its deficiency in proximal enhancers [18, 19]; its distal transcriptional regulation would further polarize MYC sensitivity to environmental stimuli [3, 18, 20, 21]. Indeed, in PCa MYC transcription responds swiftly to both AR expression levels and the availability of androgen, as evidenced by findings in AR-low LNCaP versus AR-high LNCaP-AR and VCaP cell lines [3]. Nevertheless, we initiated to crack the puzzles of androgen dual-transcriptional effects by showing the convergence of AR direct transactivation and indirect transrepression in the 8q24-MYC TAD [2, 3]. In this genomic region, AR instructs locus-wide MYC regulation by manipulating MYC-Pro distal engagements with the PCAT1 super-enhancer (SE) in the left-arm and PVT1 SE in the right arm [3, 18, 20].

Our current report was designated to define the mechanisms of androgen-elicited dual-transcriptional functions in global and local contexts. For this purpose, we focused on the 8q24-MYC locus that contains multiple AR binding sites (ABSs) in annotation with PCa-specific enhancers. Using an innovative CRISPR/Cas9-mediated genome-editing strategy, we deleted several key ABSs from the left and right arms of this TAD, respectively. The outcomes of these KO tests were consistent with the concept that MYC transcription is coupled to AR direct activation and indirect repression functions. In the end, we further took bioinformatics to systemically dissect androgen effects in the 8q24-MYC locus and along the PCa genome, demonstrating that H3K27ac and BRD4 are re-distributed from pioneer factors (FoxA1 and HoxB13) to AR sites. Collectively, our findings recognized AR-mediated transcription is intrinsically equilibrated between its dual-functions that are coupled to co-factor exchanges.

## Materials/subjects and methods

### Reagents

DHT (S4757, SelleckChem, Houston, TX, USA), G418 (10131035, Thermo Fisher, Waltham, MA, USA), Puromycin (NC9138068, Fishersci, Hampton, NH, USA), Hygromycin B (Thermo Fisher, 10687010), Blasticidin (Thermo Fisher, A1113903), Lenti-gRNA-Neo (G418) vector (LGN; Catalog. 104992, Addgene, Watertown, MA, USA) and Lenti-gRNA-Puro vector (LGP; Addgene, Catalog. 104990). Transfections were conducted using Lipofectamine 2000 (11668019, Life Technologies, Carlsbad, CA, USA), following the manufacturer’s direction.

### Antibodies

AR (Ab74272, Abcam, Waltham, MA, USA), BRD4 (A301-985A, Bethyl, Montgomery, TX, USA), H3K27ac (C15410196, DIAGENODE, Denville, NJ, USA), H3K4me1 (Abcam, ab8895), H3K4me2 (Abcam, ab7766), H3K9me1 (Abcam, ab9045), H3K9me2 (Abcam, ab1220), H3ac (Thermo Fisher, 39139), FoxA1 (Abcam, ab23738), Med1 (1710530, Sigma-Aldrich, St. Louis, MO, USA), CDK9 (Cat. sc-8338, Santa Cruz, Dallas, TX, USA), cyclin T1 (CCNT1, Santa Cruz, Cat. sc-10750), p-RNA Pol II-Ser5 (pPol2-S5, Abcam, Cat. ab5131), and IgG (2729S, Cell Signaling, Danvers, MA, USA).

### Cell lines

LNCaP cells were grown in RPMI medium containing 10% FBS and VCaP cells were grown in DMEM medium containing 10% FBS. LNCaP AR overexpression (OE) stable line (LNCaP-AR or LA) was generated with lentiviral vector expressing Flag-tagged AR and hygromycin B selection; LNCaP-AR Cas9 OE stable line (LNCaP-AR-Cas9 or LAC) was generated based on lentiviral vector expressing Cas9, under Hygromycin B and Blasticidin selection [22]. For CRISPR/Cas9-mediated genomic knock-out (KO) test, LNCaP-AR-Cas9 cell line was infected with Lenti-gRNA-Neo vector (LGN) expressing a pair of upstream sgRNAs (U1/U2) and Lenti-gRNA-Puro vector (LGP) expressing a pair of downstream sgRNAs (D1/D2), respectively. Stable pools were selected under Hygromycin B, Blasticidin, G418 and Puro. For androgen-starving conditions, cells were grown in medium containing 5% CDS (charcoal–dextran stripped FBS). DHT was used at final concentration of 10 nM unless specified otherwise.

### Chromosome conformation capture (3C) assay

The procedure was reported [3]. Briefly, VCaP cells treated with vehicle (control) or DHT (10 nM, 2 h) were fixed with 1% formaldehyde for 10 min and quenched by glycine. Cells were lysed and nuclei were resuspended in 100 μl of 0.5% sodium dodecyl sulfate (SDS). Nuclei extraction was performed by incubation at 62 °C for 10 min, followed by dilution in Triton X-100 to quench the SDS. The chromatin fraction was digested for overnight at 37 °C with 500 U of PstI (R0140S, NEB, New England Biolabs, Ipswich, MA), followed by inactivation at 80 °C for 20 min. Ligation was conducted in 1 ml volume with 8000 U of T4 DNA Ligase (NEB, M0202L) at room temperature. Proteinase K (30 μl of 20 mg/ml) (NEB, P8107S) was next added for incubation at 65 °C for overnight. RNase A (15 μl) (10 mg/ml, Thermo Fisher, EN0531) was next added for incubation at 37 °C for 45 min. Samples were then purified with phenol:chloroform:isoamyl alcohol (25:24:1; Thermo Fisher, 15593-031) extraction that was followed by ethanol precipitation. Eluted dsDNA was quantified using Qubit (Model 3.0, Life Technologies). For 3C assays based on the VCaP-PstI 3C library DNA: 100 ng as template for nest-PCR and 400 ng as template for quantitative droplet digital PCR (ddPCR) analyses, respectively.

### PCR and RT-PCR kits, primers and probes, and gRNA constructs oligos

The detailed information was included in Additional file 1. The uniqueness of all primers and oligos in this study has been validated by BLAST against human genome and transcripts.

### Taq RT-qPCR (RNA template)

The test was conducted using the Fast Virus 1-step kit [Applied Biosystems (Life Technologies), Cat. 4444434]. The TaqMan primer–probe sets were all from Life Technologies (Applied Biosystems). Primer/FAM-probe sets: MYC (Hs00153408_m1), PSA/KLK3 (Hs02576345_m1), PVT1 (Hs01069041_m1); and the CCAT1 gene TaqMan primer/FAM-probe set: forward primer: GGCCAGCCCTGCCACT; reverse primer: CAGTTTTCAAGGGATTTTAGGAGAA; and probe: ACCAGGTTGGCTCTGTATGGCTAAGCGT. The inventoried internal control is the GAPDH primer/VIC-probe set (4310884E).

### Datasets in the public domains

ChIP-Seq and RNA-Seq datasets used in this study are retrieved from the GEO database, as listed below: LNCaP AR ChIP-Seq, GSE83860 [23]; LNCaP HoxB13 ChIP-Seq, GSE96652 [24]; LNCaP FOXA1 ChIP-Seq, GSE83860; LNCaP H3K27ac ChIP-Seq, GSE51621 [25]; VCaP AR ChIP-Seq, GSE55062 [26]; VCaP HoxB13 ChIP-Seq, GSE96652; VCaP FOXA1 ChIP-Seq, GSE96652; VCaP H3K27ac ChIP-Seq, GSE157107 [3]; VCaP BRD4 ChIP-Seq, GSE55062; LNCaP-Abl shFOXA1 H3K27ac_ChIP-seq, GSE72467; LNCaP-Abl shFOXA1 RNA-seq, GSE72534; LNCaP FOXA1_Mutation H3K27ac_ChIP-seq, GSE133387; LNCaP FOXA1_Mutation RNA-seq, GSE133384; LNCaP HoxB13_Mutation H3K27ac_ChIP-seq, GSE153583; LNCaP HoxB13_Mutation RNA-seq, GSE153585. 22Rv1 Hi-C analysis was based on GEO datasets GSE118629 and PCa risk SNP alignment was based on the GWAS Catalog database.

### Bioinformatics analyses

Raw ChIP-Seq data were trimmed and then mapped to either Hg19 or Hg38 reference genome as indicated. MACS2 [27] and ucsctools were then utilized to call peaks and generate bigwig files containing normalized ChIP-Seq signal. Hi-C data were processed by Juicer pipeline [28] to generate.hic files. AR motif PWM was retrieved from R package ‘MotifDb’ and used for AR motif scan by R function ‘matchPWM’. R package ‘plotgardener’ [29] was used to visualize ChIP-Seq, Hi-C and AR motif data at 8q24 region. For RNA-Seq analysis, raw sequencing reads were first trimmed by Trim Galore and clean reads were next aligned using STAR with parameters “–outSAMattributes NH HI NM MD–outSAMstrandField intronMotif –quantMode GeneCounts”. Fragments Per Kilobase of exon model per Million mapped fragments (FPKM) were then calculated based on gene read count.

### Statistical analyses

Generally, data in bar graphs represent mean ± SD of three biological replicates and are representative of three independent experiments. Two-tailed unpaired Student’s t-test was performed to calculate the statistical significance of two independent groups. P < 0.05 was considered statistically significant: *P < 0.05, **P < 0.01, ***P < 0.001.

## Results

### Androgen-motivated VCaP transcriptomes are coupled to AR direct and indirect dual functions

**Fig. 1:**
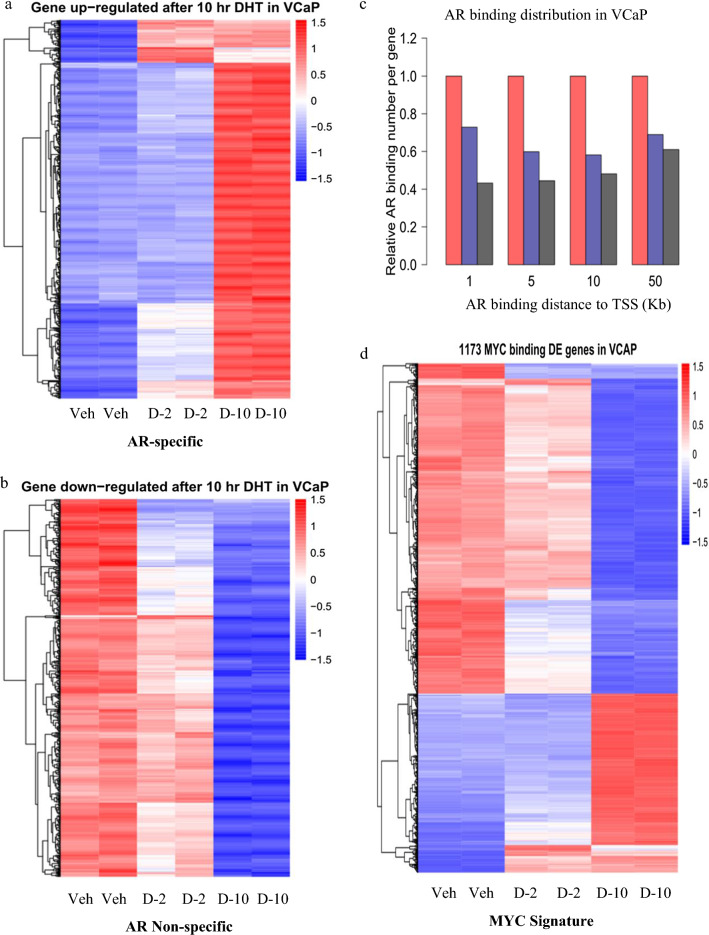
Androgen-motivated VCaP transcriptomes are coupled to AR direct and indirect dual-functions. **A**, **B** Heatmap analyses of androgen-activated versus androgen-repressed transcriptomes of VCaP treated with androgen (Vehicle control versus 10 nM of DHT for 2 h and 10 h, respectively) (GSE157107). **C** Global AR binding distribution analyses: chromatin affinity and proximity were computed based on VCaP AR ChIP-seq datasets GSE55062. Color denotes: androgen-activated genes (red); androgen-repressed genes (blue); and genomic background (black). **D** Bioinformatics analysis of androgen-mediated RNA-Seq in combination with MYC ChIP-Seq datasets (GSE157107)

Recent advancements on chromatin techniques revealed the bi-directionality of AR-mediated transcription: transactivation versus transrepression [1–3]. Consistent with the linkage of AR dual-functions to co-factor re-distribution, AR stimulatory and repressive activities are temporally coupled, as evidenced by co-incidence in up-regulated versus down-regulated VCaP transcriptomes along the time-course of DHT treatment (Fig. 1A, B). Indeed, AR activation function is specifically linked to chromatin affinity while its repression activities are less dependent on DNA-binding (Fig. 1C) [2, 3]. Of importance, the non-specificity in co-factor re-distribution underlies AR indirect effects, as exemplified by the extension of androgen effects into the MYC-occupied gene subsets (Fig. 1D). Interestingly, AR activated and repressed programs were both enclosed in MYC signatures, with higher enrichment in repressed genes (Fig. 1D). These findings are consistent with reports that AR and MYC transcriptional networks are mutually repressive and linked by co-factor competition [3, 15, 16]. The hyper-sensitivity of the MYC gene to environmental stimuli is derived from its short half-life in both mRNA and proteins and its regulation by distal enhancer [3]. Here we exploited the prominent 8q24-MYC locus that confers intensified androgen responsiveness to explore AR bi-directional functions.

### The 8q24-MYC locus is clustered with AR binding peaks in annotation with hallmarks of enhancer and distinct androgen responsiveness

**Fig. 2:**
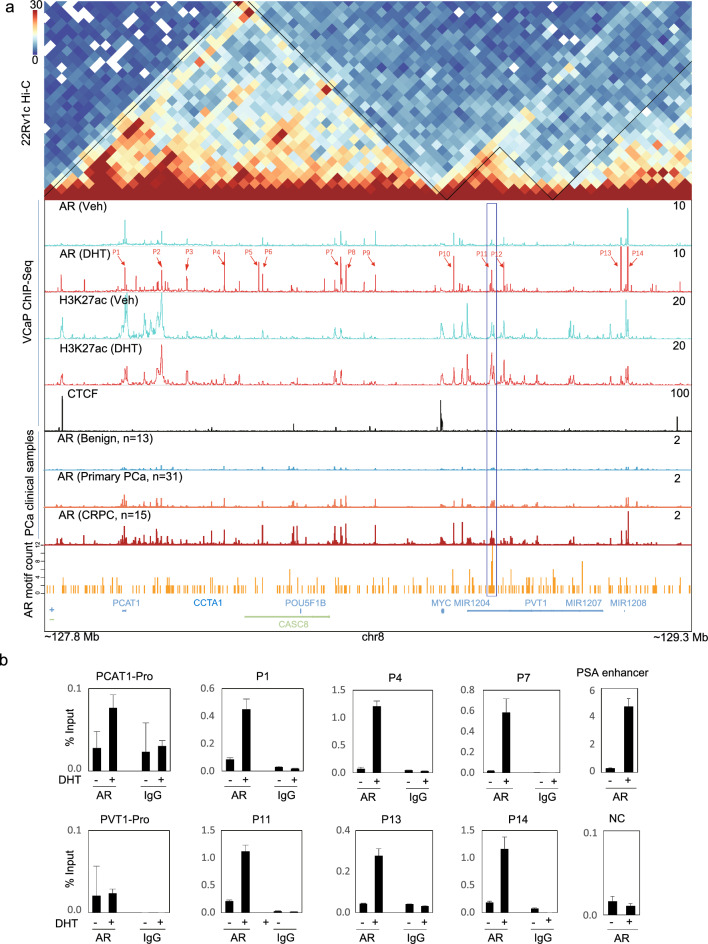
In PCa cells, the 8q24-MYC locus is clustered with AR binding peaks that were in annotation with enhancer hallmarks and distinct androgen responsiveness. **A** Annotation of 8q24-MYC locus with 22RV1 HiC, VCaP AR ChIP-Seq, VCaP H3K27ac ChIP-Seq, VCaP CTCF ChIP-Seq, clinical AR ChIP-Seq (benign, primary PCa, and CRPC) and AR motif scan. A CTCF peak right on the MYC-Promoter borders two TADs in left and right arms of this locus, respectively. P1–P14: major AR binding peaks in the 8q24-MYC locus that were highlighted and aligned from centromeric to telomeric directions. Also highlighted was the P11 site that contains multiple AR binding motifs. **B** VCaP cells in CDS medium were treated with 10 nM of DHT for 4 h, followed by AR ChIP-qPCR analysis of randomly selected AR binding sites (ABSs) as normalized to input. PSA enhancer (PSA-Enh) was used as positive control while NC (negative control) is an AR-irrelevant chromatin site. Data are mean values ± SD for three biologically independent samples

The prominent 8q24-MYC locus is a gene desert featured in residency of MYC and flanking lncRNAs and multiple prostate risk regions [3, 20]. Mechanistically, androgen elicits locus-wide repression through co-factor competition that depends on AR nuclear entry but not chromatin-binding activities [3]. However, androgen responsiveness was distinct among genes and ABSs (P1-P14) in this locus (Fig. 2A) [3], indicating the engagement of both global and local effects. Interestingly, in PCa cells MYC left and right arms in this locus were isolated as two CTCF-bordered TADs, consistent with our previous report (Fig. 2A) [3]. Furthermore, annotation of VCaP and clinical AR binding profiles in this locus indicated each ABS was associated with distinct enhancer status, as marked by H3K27ac occupancy (Fig. 2A). By motif scan, we identified AR binding motifs (ABMs) in this locus, with particularly high enrichment at the P11 site. We also performed ChIP-qPCR to have randomly verified AR enrichment on these AR binding peaks (Fig. 2B). In contrast, there was no or minimal AR occupancy at the PCAT1-promoter (Pro), PVT1-Pro and negative control (NC) sites. Additionally, as compared to AR binding at the PSA-enh (a classic and robust ABS), its affinity with the 8q24 locus is about 4–15 folds lower, indicating relative weak association that generally featured AR-repressed loci [2, 3].

### An AR-binding site (P10) ~ 25 Kb downstream of the MYC gene was androgen-induced for interaction with MYC-promoter and epigenetically repressed by androgen

**Fig. 3:**
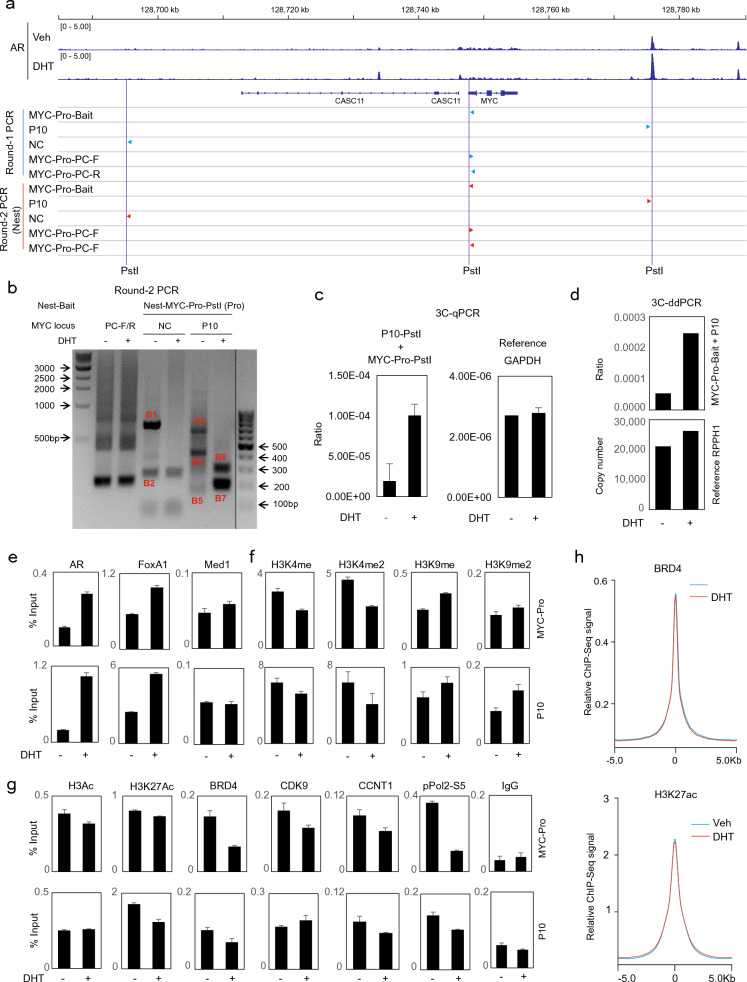
An AR-binding site (P10) ~ 25 Kb downstream of the MYC gene was androgen-induced for interaction with MYC promoter and epigenetically repressed upon androgen treatment. **A** Schematic showing of the MYC-P10 locus in alignment with VCaP AR ChIP-Seq tracks, the 3C assay strategy based on PstI digestion (the targeting bait (anchor), relevant PstI sites, control sites and PCR primers). **B** Two-round PCR (nest-PCR) assay was performed to amplify the 3C signals of MYC-Pro-P10 hybrids, shown here was the round-2 PCR result. Highlighted DNA bands (B1–B7) were excised and purified for DNA-Seq, confirming androgen-induced MYC-Pro-P10 interaction in the B6 and B7 bands that are derived from two P10-proximal PstI sites. PC-F/R: positive loading control primers that target the MYC-Pro locus without PstI site in the amplicon. NC: negative control primer that targets a distal Chr8 site with proximal PstI site but no AR binding. **C** RT-qPCR analysis of the 3C signal between MYC-Pro and P10, with GAPDH (no PstI site in its amplicon) as reference. Data are mean values ± SD for two biologically independent samples. **D** 3C-ddPCR analysis of MYC-Pro-P10 interaction. The copy number reference is an RPPH1 probe that does not contain PstI site in the amplicon. **E**–**G** VCaP cells in androgen-depleted medium were treated with 10 nM of DHT for 4 h and then subjected to ChIP-qPCR analysis. Occupancy at the MYC-Pro and P10 sites are assessed by RT-qPCR that was normalized to input. Data are mean values ± SD for three biologically independent samples. **H** Global calculation of genomic BRD4 and H3K27ac chromatin occupancy peak counts based on the GSE55062 datasets. See Additional file 1: Fig. S1–S3 for additional information

MYC is known to have no proximal enhancers and depends on distal elements for transactivation [18, 19], and to find the nearest enhancer we next sought ABSs in vicinity of the MYC gene body. For this purpose, we focused on P10, an AR binding peak ~ 25 Kb downstream of the MYC gene that had modest AR (but not MYC) affinity (Fig. 3A) [3]. As shown in Fig. 2A, P10 is marked by an intermediate H3K27ac peak, indicating it is a modest enhancer. Our previous report showed MYC-Promoter (MYC-Pro) was looped with a series of enhancers within the 8q24 region, including P10 [3]. Here we validate these findings by conducting 3C-nest-PCR, 3C-qPCR, and 3C-ddPCR assays, together with relevant controls (Fig. 3B–D and Additional file 1: Fig. S1–S3). Consistently, 3C-qPCR and 3C-ddPCR analyses both affirmed androgen-dependent MYC-Pro-P10 interaction. In the nest-PCR assay we included both loading control and negative control site (Fig. 3B and Additional file 1: Fig. S1), and androgen specifically induced two MYC-Pro-P10 hybrids that are based on fusion with two P10-proximal PstI sites, respectively (Fig. 3B). The identities of these two bands (B6–B7) were validated by DNA purification, sequencing, and alignment; while additional bands (B1–B5) are non-specific (Fig. 3B, Additional file 1: Figs. S2, S3).

To further expose the mechanisms of androgen-mediated MYC transrepression, we monitored epigenetics of the MYC-Pro and P10 sites in DHT-treated VCaP with ChIP-qPCR analyses. At both sites, androgen stimulated AR and FoxA1 occupancy, enhanced the transrepressive markers H3K9me1 and H3K9me2, and repressed the transactive markers H3K4me1 and H3K4me2 (Fig. 3E, F). Consistent with the repressive effects, androgen down-regulated H3ac, H3K27ac, and BRD4 binding, which is associated with decline in the pTEFb (CDK9/CNNT1) complex and RNA PolII activity (as marked by p-Pol2-S5) (Fig. 3G). In our previous report, H3K4me2 and p-Pol2-S5 were similarly used to mark ABS activation on multiple gene promoters [2]. Importantly, the decrease in H3K27ac and BRD4 binding to MYC-Pro and P10 sites was a local effect, as their global chromatin occupancy was barely decreased by androgen (Fig. 3H). Together, these observations confirmed epigenetics repression of MYC proximal elements by androgen in VCaP.

### CRISPR/Cas9-mediated genomic knock-out (KO) of P10 to evaluate its impact on androgen-elicited MYC transrepression

**Fig. 4:**
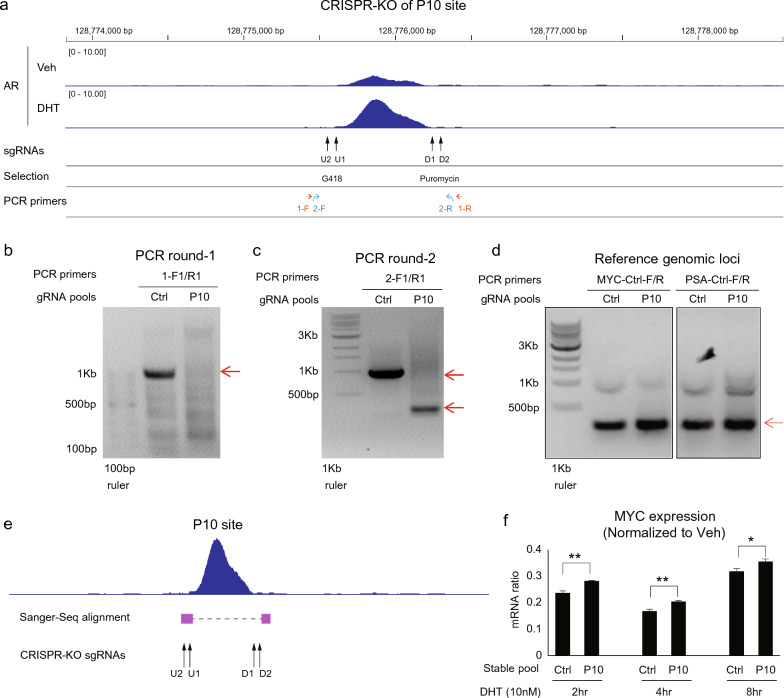
CRISPR/Cas9-mediated genomic knock-out (KO) of P10 to evaluate its impact on androgen-elicited MYC transrepression. **A** A schematic showing of the MYC gene locus with the P10-KO strategy. Highlighted are VCaP AR ChIP-Seq tracks, two pairs of P10 targeting gRNA (U2/U1 for upstream gRNAs under G418 selection; and D1/D2 for downstream gRNAs under Puro selection), together with PCR and DNA-Seq primers. **B** Genomic DNA of stable pools were amplified by round-1 PCR. The predicted PCR product sizes: control pool (995 bp) and P10-KO pools (371 bp, 317 bp, 313 bp, and 259 bp). **C** The round-1 PCR products were re-amplified by round-2 PCR (nest-PCR). The predicted PCR product sizes: control pool (941 bp), P10-KO pools (316 bp, 262 bp, 258 bp, and 204 bp). **D** as loading controls, control PCR tests were conducted with indicated primers to amplify the MYC and PSA loci that are not targeted by gRNA. Arrow heads indicated bands with expected sizes. **E** The identities of the major nest-PCR bands were assessed and verified by band excision, DNA purification, DNA-Seq, and alignment. The results indicated that editing predominantly occurred at U1 and D2 gRNA sites. **F** Control (C) and P10-KO pools in androgen-depleted medium were subjected to 10 nM of DHT treatment for indicated time points. TaqMan RT-qPCR was conducted on MYC mRNA expression (GAPDH as internal reference). The normalized read-outs were shown, with the vehicle-treated control samples being set at 1. Data are mean values ± SD for three biologically independent samples. P values are two-sided Student’s t test. *P < 0.05; **P < 0.01. See Additional file 1: Fig. S4, S5 for additional information

To further dissect P10 functions in androgen-induced MYC transrepression, we designated a novel CRISPR/Cas9-mediated genome-editing approach, aiming to enhance KO efficiency. Specifically, lentiviral vectors were constructed to excise the P10 site by co-expressing an upstream gRNA pair (U1/U2) under G418 selection and a downstream gRNA pair (D1/D2) under Puro selection, respectively (Fig. 4A). Lentiviral vectors bearing a pair of non-specific gRNAs were used as control. For our objectives, we employed AR-overexpression LNCaP cells (LNCaP-AR, or LA) that resembled androgen effects in AR-high VCaP cells and readily allowed ectopic Cas9 expression [3, 22]. LNCaP-AR-Cas9 (LAC) cells were next infected with equal molar of paired gRNA vectors for selection, to generate stable pools. Then we performed nest-PCR tests based on the genomic DNA of stable pools to evaluate the outcomes of editing with primers flanking the target site, with two independent genomic loci as loading controls (Fig. 4A–D). As compared to the single band in the control pool, genome-editing based on two pairs of gRNA produced multiple amplicons in the P10 stable pool at smaller and varied sizes (Fig. 4C). The variations in the size and density of DNA bands in the P10 sample reflected distinct editing efficacy among various gRNA combinations. In any case, the results demonstrated paucity of the wild-type PCR band in the P10-KO pool, affirming that a majority of the cell population became deficient in the P10 site. Consistently, the identities of these amplicons were validated by DNA purification and sequencing, with the followed alignments verifying KO occurred predominantly at the cleavage site of U1 and D2 gRNAs (Fig. 4C, E and Additional file 1: Figs. S4, S5).

Next, the control and P10 pools were subjected to functional assay, to assess the effects of P10-KO on androgen-mediated MYC repression. Stable pools in androgen-depleted medium were subjected to 10 nM of DHT treatment for indicated time points, followed by TaqMan RT-PCR analysis of total RNA to examine MYC expression (GAPDH as internal reference). As shown in Fig. 4F, normalized read-outs demonstrated P10-KO slightly lessened MYC transrepression by androgen.

### KO of ABSs in the PVT1 and PCAT1 loci to assess effects on androgen-elicited MYC repression

**Fig. 5:**
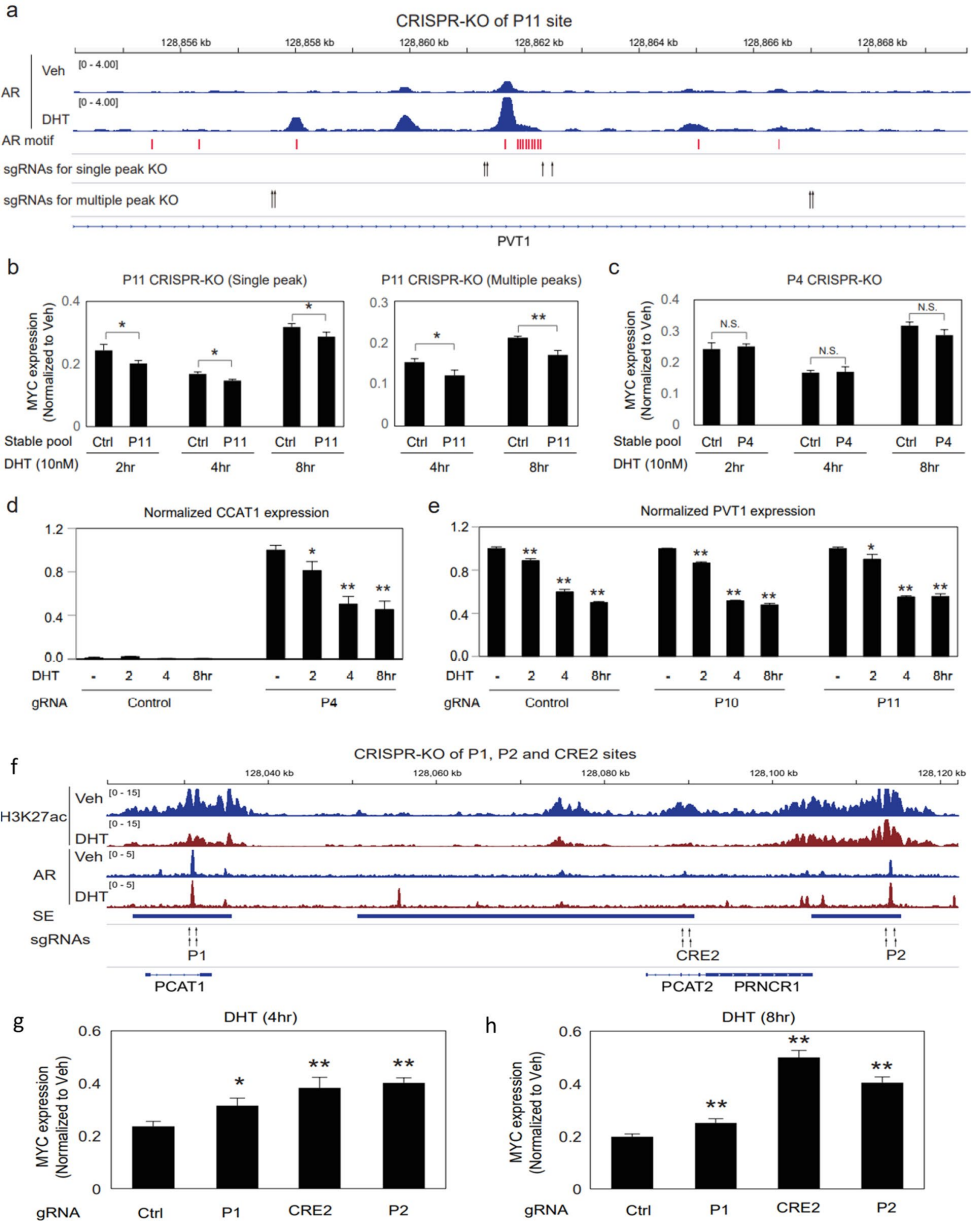
KO of ABSs in the PVT1 and PCAT1 loci to assess effects on androgen-elicited MYC repression. **A** A schematic showing of the P11 site KO strategy. Highlighted are VCaP AR ChIP-Seq tracks, two pairs of gRNA targeting the central AR peak versus two pairs of gRNA targeting the all 5 major AR peaks, respectively. Also highlighted was a cluster of AR binding motifs enriched in the P11 site, based on motif scan analysis. **B** Control and P11-KO (central AR peak KO, left panel; multiple AR peaks KO, right panel) stable pools in androgen-depleted medium were subjected to 10 nM of DHT treatment for indicated time points. TaqMan RT-qPCR was conducted on MYC mRNA expression (GAPDH as internal reference), followed by data normalization with the control (vehicle) being set at 1. **C** Similarly, control and P4-KO stable pools in androgen-depleted medium were subjected to 10 nM of DHT treatment for indicated time points. TaqMan RT-qPCR was conducted on MYC mRNA expression (GAPDH as internal reference), followed by data normalization with the control (vehicle) being set at 1. **D** total RNA of the control and P4-KO pools was assessed by TaqMan RT-qPCR for the expression of the CCAT1 gene that is in proximity to the P4 site. **E** Total RNA of the control, P10-KO and P11-KO (central AR peak KO) pools was assessed by TaqMan RT-qPCR for expression of the PVT1 gene that is in proximity to both P10 and P11 sites. **F** Schematic showing of the PCAT1 SE that was in annotation with VCaP AR and H3K27ac ChIP-Seq tracks. This locus contains three lncRNAs (PCAT1, PCAT2, and PRNCR1) and several AR and H3K27ac enhancer peaks. Also highlighted was the editing strategy, aiming to excise the P1, P2, and CRE2 sites. **G**, **H** Control and 3 specific KO pools in androgen-depleted medium were subjected to 10 nM of DHT treatment for indicated time points. TaqMan RT-qPCR was conducted on MYC mRNA expression (GAPDH as internal reference), followed by data normalization with the control (vehicle) being set at 1. Data are mean values ± SD for three biologically independent samples. P values are two-sided Student’s t test. *P < 0.05; **P < 0.01. N.S., no statistical significance. See Additional file 1: Figs. S6, S7 for additional information

We next similarly targeted an ABS (P11) in the MYC 3′ flanking lncRNA PVT1 region that is enriched in AR binding motifs (ABMs) (Fig. 2A). Consistent with the findings in breast cancer that PVT1 promoter and enhancers engage in MYC regulation, our group recently reported that in PCa MYC-Pro distally interacts with PVT1 locus enhancers [3]. Specifically, androgen elicited MYC-Pro docking with PVT1-SE at the expense of PCAT1-SE. Although androgen attenuated the engagement of MYC-Pro with majority of enhancers in the 8q24-MYC locus, P4 on the left arm and P11 on the right arm are among the few sites that were actually motivated by androgen for both AR and H3K27ac occupancy (Figs. 2A, 5A) [3]. A closer view of P11 further indicated it actually contains 5 AR sub-peaks, with the central peak being clustered with at least 10 potential ABMs, making it one highly ABM-enriched locus along the VCaP genome (Fig. 5A, Additional file 1: Fig. S6). Next, we similarly took the CRISPR/Cas9-mediated KO approach to evaluate the functions of P4 and P11 on androgen-mediated MYC transrepression. At both sites double gRNA pairs rendered high editing efficiency, as validated by nest-PCR amplification and DNA-seq alignment (Additional file 1: Figs. S4, S6, S7).

The control versus KO pools in androgen-depleted medium were then subjected to DHT treatment as indicated, followed by TaqMan RT-PCR assay for MYC expression (Fig. 5B–E). As shown in the normalized read-outs (Fig. 5B), P11-KO slightly deepened MYC transrepression by androgen. To verify this finding, we next took a rather radical approach to KO all 5 AR sub-peaks in the P11 site (Fig. 5A, B); and gene expression analyses consistently showed KO of multiple peaks also slightly deepened MYC transrepression by androgen. In contrast, P4-KO had no pronounced effects on MYC messages, in accordance with our previous HiChIP finding that P4 site has no distal interaction with MYC-Pro (Fig. 2A) [3]. Together, these findings suggested AR on the P11 site was activated by androgen to neutralize its repressive effects on MYC, attesting the convergence of and equilibration between AR dual-functions on a single site. Considering proximity of the P4 site to the CCAT1 gene, we next assessed the effects of P4-KO on its transcripts. Significantly, P4-KO enhanced basal CCAT1 expression for ~ 100-fold; but however, did not reverse its repression by androgen that was reported in both VCaP and LNCaP-AR cells (Fig. 5D) [3]. In addition, KO of both P10 and P11 did not pronouncedly alter androgen-elicited transrepression of their proximal PVT1 gene expression either, although androgen repressed PVT1 gene (on the right arm) at less extent than other genes (on the left arm) in the 8q24 locus (Fig. 5E) [3].

Previous report established the PCAT1 SE as a robust developmental and locus-wide enhancer that distally engaged with MYC-Pro [3, 20]. We also have demonstrated that SEs in general are extremely sensitive to androgen elicitation and PCAT1 SE is among the top SEs highly prone to androgen repression [3]. In accordance, an enhancer site (CRE2) within this area has been showed to activate MYC transactivation in VCaP cells under the setting of transient genome-editing [30]. We have evidenced that ABS-associated enhancers on left arm of this locus are prostate-specific; indeed, chromatins at these sites were active in AR(+) LNCaP and VCaP but not in AR(−) PC3 and DU145 cell lines (Additional file 1: Fig. S8) [3]. Notably, this left arm region is also featured by enrichment of clinical PCa risk SNPs (single nucleotide polymorphisms), with particular concentration on the PCAT1 SE that is co-occupied by AR co-factor FoxA1 and HoxB13 (Additional file 1: Fig. S9) [3]. Based on these findings, we speculated KO of key regulatory elements in PCAT1 SE would render an impact on androgen-elicited MYC transrepression. Considering the massive size of the PCAT1 SE, we similarly took the above KO method to delete P1, P2, and CRE2 sites, all having pronounced H3K27ac occupancy (Figs. 2A and 5F). Next the control and KO pools were treated with androgen as indicated in androgen-depleted medium (Fig. 5G, H). Significantly, TaqMan RT-PCR analyses showed that in all 3 KO pools MYC transrepression by androgen was slightly relieved (Fig. 5G, H). These findings are consistent with the notion that unliganded AR are reprogrammed to occupy this SE, priming it for transactivation. They also suggest that PCAT1 SE affects androgen-elicited MYC transrepression in a mechanism that is similar to that of the P10 site but distinct to the P11 site, in alignment with P11 alone being an androgen-motivated strong enhancer mediating MYC-Pro looping and transactivation [3].

### Genome-wide and site-specific validation of co-factor equilibration between AR dual-functions

**Fig. 6:**
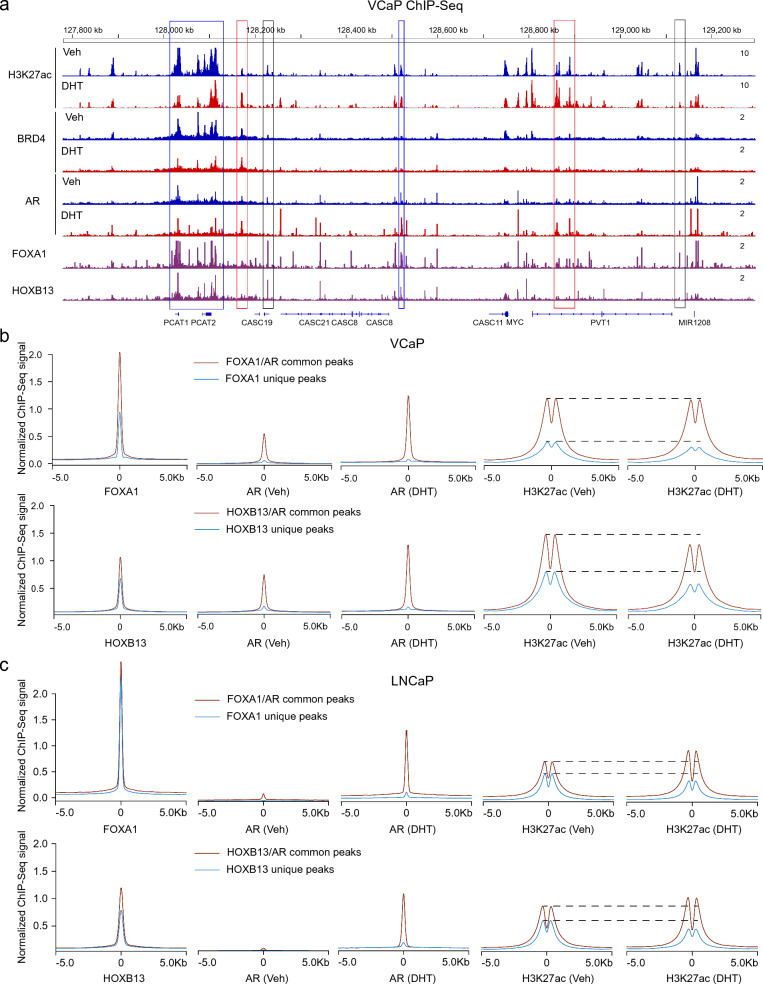
Genome-wide and site-specific validation of co-factor equilibration between AR dual-functions. **A** Annotation of 8q24-MYC locus with VCaP AR ChIP-Seq, H3K27ac ChIP-Seq, BRD4 ChIP-Seq, FoxA1 ChIP-Seq and HoxB13 ChIP-Seq. Blue rectangle frames: AR sites, strong FOXA1/HOXB13 binding, no H3K27ac/BRD4 increase after DHT treatment; Red rectangle frames: AR sites, week FOXA1/HOXB13 binding, H3K27ac/BRD4 increase after DHT treatment; Black rectangle frames: Week/No AR binding, strong FOXA1/HOXB13 binding, H3K27ac/BRD4 decrease after DHT treatment. **B**, **C** Global co-factor re-distribution profiling of VCaP and LNCaP ChIP-Seq datasets, aiming to display androgen effects on AR, FOXA1, and HOXB13 binding sites upon androgen treatment. Specifically, FOXA1/HOXB13 sites were divided into FOXA1/HOXB13-unique and FOXA1/HOXB13-AR common sites; and these two sets responded distinctly to androgen. See Additional file 1: Fig. S11 for additional information

As aforementioned, co-factor re-distribution intrinsically couples with androgen-elicited transcriptional activation and repression. Specifically, each individual androgen-elicited gene and chromatin site exhibited distinct responsive profiling; for example, the magnitude and duration of androgen responsiveness are different among genes in the left arm, middle section, and right arm of the 8q24-MYC locus [2–4]. Based on these observations, we reason that the transcriptional outcome is a sum of global and local androgen effects, as evidenced in our KO tests. Consistently, annotation of the 8q24-MYC locus along Hg19 and Hg38 references both indicated the association of each enhancer site with distinct spectrums of transcriptional factor and androgen responsiveness (Fig. 6A; Additional file 1: Fig. S10). For instance, FoxA1/HoxB13-primed sites (no AR) are massively repressed by androgen, while on AR-occupied sites H3K27ac signals correlated with AR binding statuses prior and posterior androgen treatment. Generally, sites with net increase in AR binding had sustained or enhanced H3K27ac signals, in accordance with the direct role of AR as a transactivator.

To generalize our findings along the PCa genome, we next took global bioinformatics strategy on VCaP datasets. For this purpose, we focused on FoxA1 and HoxB13, two key pioneering factors in PCa AR reprogramming [31, 32]. By globally clustering chromatin-binding profiles, we found that AR actually could program FoxA1 chromatin binding, as evidenced by increased FoxA1 affinity in FoxA1-AR common sites over FoxA1-unique sites (Fig. 6B). Androgen also degraded the enhancer status (as marked by H3K27ac) on FoxA1-unique sites but not FoxA1-AR common sites. Similar observations occurred at the HoxB13 sites as well, with the exception that H3K27ac signals got attenuated by androgen on HoxB13-AR common sites at less extent than HoxB13-unique sites (Fig. 6B). We similarly analyzed AR-low LNCaP cells, in which androgen exhibited modest and transient repressive effects [2, 3]. As shown (Fig. 6C), androgen decreased H3K27ac expression at both FoxA1-unique and HoxB13-unique sites; while the signals were all increased at AR common sites.

The above findings supported that androgen elicited indirect repressive effects by transferring co-activators from FoxA1 and HoxB13 sites to AR sites. To affirm these findings, we next systemically addressed chromatin occupancy of these factors at AR-binding sites along the VCaP genome. As shown, DHT impacted negatively basal-AR binding affinity but positively liganded-AR binding affinity; similar trends were noted on H3K27ac binding affinity at these AR sites, consistent with re-distribution of H3K27ac from androgen-repressed to androgen-activated AR sites (Additional file 1: Fig. S11A, B). Significantly, inverse correlation in chromatin occupancy occurred between both FoxA1 and HoxB13 versus AR and H3K27ac (Additional file 1: Fig. S11C–F). Collectively, these findings were in agreement with observations in Fig. 6, all indicating androgen-motivated migration of H3K27ac from FoxA1 and HoxB13 sites to AR sites.

To further extend the translational relevance of our findings, we next examined effects of FoxA1 and HoxB13 manipulations on the 8q24-MYC locus activation and MYC gene expression in AR(+) PCa cell lines. As shown in the androgen-refractory LNCaP-abl cells, FoxA1 KD by siRNA had repressive effects on ABS enhancers (particularly the PCAT1 SE) on left arm of this locus and MYC transcription (Additional file 1: Fig. S12A, B). In contrast, in the androgen-sensitive LNCaP stable cell lines FoxA1 forkhead domain mutants (FKHD-MSs: D226G and M253K) activated ABS enhancers (particularly the PCAT1 SE) on the left arm and increased MYC messages (Additional file 1: Fig. S12C, D). We also analyzed an LNCaP datasets that are based on siRNA KD of endogenous HOXB13 in conjunction with ectopic expression of HOXB13 WT versus G84E mutant, showing mutation-driven modest activation of ABS enhancers (including the PCAT1 SE) on the left arm and modest increase in MYC transcripts (Additional file 1: Fig. S12E, F). Together, these findings supported mechanistic linkages between locus-wide co-factor distribution and transcriptional regulation of the MYC gene.

## Discussion

The bi-phasic effects of androgen in PCa have been recognized for decades without mechanistic insight; however, recent advancements in chromatin and epigenetics techniques enabled the conceptual revision of AR functionalities. AR still remains as a predominant transactivator, however, its transcriptional effects have been massively oversimplified. Reports in recent 10-years clearly evidenced AR dual-functions: direct transactivation versus indirect transrepression. As compared to its chromatin-mediated transactivation, AR repression function is indirect and dissociated with chromatin-occupancy [1–3]. The hidden AR dual-transcriptional mechanisms have perplexed PCa field for many years but could well underly the intriguing biphasic androgen effects. We currently view AR-mediated transcription as constant dynamics, a process of active equilibration between activation versus repression factors. Indeed, we found H3K27ac and BRD4 globally migrate from pioneer factors (FoxA1 and HoxB13) to androgen-motivated AR sites upon androgen stimulation. This novel AR recognition provides rationales to the bipolar androgen therapy (BAT), in which androgen levels are polarized in extremes to either minimize AR activation or maximize AR repression.

The conversion of AR from a differential factor in normal prostate cells into a growth accelerator in PCa cells has been hypothetically linked to gained MYC regulation [14]. With chromatin and epigenetic tools, we identified AR as a locus-wide regulator of the oncogenic 8q24-MYC TAD [3, 10–12]. As we showed, both AR and MYC have activation and repression dual-transcriptional functions. Significantly, AR and MYC pathways are inversely paired in PCa cells due to co-factor competition; and their partial signature compensation would safeguard essential transcriptional programs and cellular activities during functional transition. The sensitivity of the MYC gene to transcriptional regulation is intensified by its short half-life in both mRNA and proteins and its distal regulation by SEs that are vulnerable to environmental perturbation [3, 18–21, 33]. Taken advantages of their androgen hyper-sensitivity, MYC and the 8q-24 locus were singled out in current report to validate AR bi-directional regulation. Considering the massive scales of both AR and MYC transcriptomes, we conceive the spectral exchanges between their signature programs would have global impacts on co-factor profiles.

In accordance with the topological property of 8q24 region in PCa, MYC-Pro chromatin interactions exclusively occur within this locus [3]. In the current report, we focused on several AR-occupied enhancers sites in this region. AR activation functions on MYC were evidenced by the findings that JQ1 inhibits MYC expression in AR(+) but not AR(-) PCa cell lines [26], AR binds to developmental enhancers in this locus, AR and MYC correlate in PCa expression, and AR wild-type represses MYC at lesser extent in comparison to its mutant counterparts deficient in DNA binding [3]. These observations are in accordance with AR classic functions as a transactivator that interacts predominantly with co-activators. Indeed, our KO tests determined that the P11 site (with minimal or no basal AR binding) functions to rescue MYC transcription from androgen-elicited repression; while multiple ABSs in the PCAT1 SE (with robust AR basal binding) function to sustain basal MYC expression. Consequently, KO of ABSs in P11 versus PCAT1 SE sites confer distinct effects to androgen-elicited transrepression. These observations further argue for the direct activation role of AR at the P11 site that is in equilibration with androgen-elicited indirect repressive functions. In contrast, the KO of P4 (a weak enhancer) led to robust expression of its neighbor gene CCAT1, consistent with the paucity of strong enhancer in the middle section of the 8q24 locus.

Androgen-mediated MYC regulation engaged with the exchange of multi-factors and the convergence of AR dual-functions and the non-specificity of AR-mediated MYC transrepression was evidenced by its dependence on AR nuclear localization but not DNA binding [3]. In current study, genome-wide analyses indicated that the transcriptional networks of both AR and MYC spread beyond their direct targets, attesting dual-functionality as an innate hallmark of transcription. Indeed, the distinct activation profiling of each individual site in the 8q24 locus reflects its dynamics between direct and indirect androgen effects. For instance, androgen did not decrease global BRD4 chromatin association, but it did so on a majority of enhancer sites in the 8q24 region that have relatively weak AR occupancy. Furthermore, genomic KOs in current study conferred minor effects rather than full reversal of androgen-elicited MYC repression. Here we reconcile these findings by generalizing AR-mediated transcription as an equilibration between its dual-functions; the transcriptional outcome at any given site is a sum of global and local androgen effects. Nevertheless, considering the limited impact of an individual ABS on MYC expression, we do not envision that manipulation of either MYC or AR in each ABS-KO clone would confer marked cellular effects in tissue culture and animal models.

It should also be emphasized that the physiological significance of the indirect AR repressive functions is non-trivial. Under basal conditions AR and MYC are both overexpressed and enriched on SEs, which in turn would sensitize androgen responsiveness [3]. In the physiological settings, human PCa frequently displays genomic amplification in the AR and 8q24-MYC loci [34–37]; in contrast, genomic loss was frequently observed on AR-activated TFs, such as the NKX3-1 and PLZF [35, 38, 39]. Based on our findings, clinical BAT reference can be connected to the expression and mutation profiles of AR, MYC, and co-factors (such as FoxA1 and HoxB13). Our findings of risk SNP enrichment in ABS regions of the 8q24-MYC locus and effects of FoxA1/HOXB13 manipulation on locus activation and MYC expression substantially extended clinical and translational significance. Together, these findings underscored the rationales to leverage more research resources to further define key co-factors bound to AR/MYC-mediated enhancers and exchanged during AR dual-functional transitions.

## Significance

Genomic KO analyses of AR-occupied enhancer sites in the 8q24-MYC locus exposed transcriptional equilibration between its dual-functions, which are mechanistically coupled to co-factor re-distribution between AR and pioneer factor FoxA1 and HoxB13.

### Supplementary Information

**Additional file 1.** **Fig.S1**. 3C analyses of the chromosomal interactions between MYC promoter and P10 in VCaP cells. **Fig.S2**. Annotation of MYC-Pro and P10 sites with 3C assay features. **Fig.S3**. DNA-Seq validation of DHT-induced MYC-Pro-P10 looping. **Fig.S4**. PCR and DNA-Seq validation of CRISPR/Cas9-mediated genomic KO of P10, P11 and P4 sites. **Fig.S5**. Annotation of the P10 site with CRISPR/Cas9 test features and DNA-Seq validation of genome-editing outcome in the P10-KO stable pool. **Fig.S6**. Annotation of the P11 site with CRISPR/Cas9 test features and DNA-Seq validation of genome-editing outcome in the P11-KO stable pool. **Fig.S7**. Annotation of the P4 site with CRISPR/Cas9 test features and DNA-Seq validation of genome-editing outcome in the P4-KO stable pool. **Fig.S8**. Alignment H3K27ac occupancy in the 8q24-MYC gene locus of typical PCa cells. **Fig.S9**. Annotate 8q24-MYC gene locus with prostate cancer risk SNP. **Fig.S10**. Re-annotate Fig.6A datasets based on the Hg38 reference. **Fig.S11**. Bioinformatics analyses of global androgen effects on distribution of AR, H3K27ac, FoxA1 and HoxB13 in VCaP cells. **Fig.S12**. FoxA1 and HoxB13 manipulation in AR(+) PCa cells impacted 8q24-MYC gene locus activities and MYC gene expression. Additional information on materials and methods

## Data Availability

Materials described in the manuscript, including all relevant raw data, will be freely available to any researcher wishing to use them for non-commercial purposes, without breaching participant confidentiality.
