# Supplementary material for: Dissecting transcription of the 8q24-MYC locus in prostate cancer recognizes the equilibration between androgen receptor direct and indirect dual-functions
Source: J Transl Med. 2023 Oct 12;21:716. doi: 10.1186/s12967-023-04429-4 (PMC10571316; doi:10.1186/s12967-023-04429-4)
Supplement: Supplementary file 1 — Additional file 1. Fig.S1. 3C analyses of the chromosomal interactions between MYC promoter and P10 in VCaP cells. Fig.S2. Annotation of MYC-Pro and P10 sites with 3C assay features. Fig.S3. DNA-Seq validation of DHT-induced MYC-Pro-P10 looping. Fig.S4. PCR and DNA-Seq validation of CRISPR/Cas9-mediated genomic KO of P10, P11 and P4 sites. Fig.S5. Annotation of the P10 site with CRISPR/Cas9 test features and DNA-Seq validation of genome-editing outcome in the P10-KO stable pool. Fig.S6. Annotation of the P11 site with CRISPR/Cas9 test features and DNA-Seq validation of genome-editing outcome in the P11-KO stable pool. Fig.S7. Annotation of the P4 site with CRISPR/Cas9 test features and DNA-Seq validation of genome-editing outcome in the P4-KO stable pool. Fig.S8. Alignment H3K27ac occupancy in the 8q24-MYC gene locus of typical PCa cells. Fig.S9. Annotate 8q24-MYC gene locus with prostate cancer risk SNP. Fig.S10. Re-annotate Fig.6A datasets based on the Hg38 reference. Fig.S11. Bioinformatics analyses of global androgen effects on distribution of AR, H3K27ac, FoxA1 and HoxB13 in VCaP cells. Fig.S12. FoxA1 and HoxB13 manipulation in AR(+) PCa cells impacted 8q24-MYC gene locus activities and MYC gene expression. Additional information on materials and methods [file 12967_2023_4429_MOESM1_ESM.docx]

**Supplementary information**

*Title*

**Dissecting transcription of the 8q24-MYC locus in prostate cancer recognizes the equilibration between androgen receptor direct and indirect dual-functions**

Ju Guo^1*^, Zhao Wei^2*^, Tianwei Jia^3,4,5*^, Liyang Wang^6,7^, Nuosu Nama^6,8^, Jiaqian Liang^9^, Xinghua Liao^10^, Xiaming Liu^11^, Yanfei Gao^12^, Xiaoqiang Liu^1^, Keshan Wang^6,13,14#^, Bin Fu^1#^, Shaoyong Shawn Chen^1,6#^

**Additional File 1, Fig.S1.** 3C analyses of the chromosomal interactions between MYC promoter and P10 in VCaP cells. **Fig.S2.** Annotation of MYC-Pro and P10 sites with 3C assay features. **Fig.S3.** DNA-Seq validation of DHT-induced MYC-Pro-P10 looping. **Fig.S4.** PCR and DNA-Seq validation of CRISPR/Cas9-mediated genomic KO of P10, P11 and P4 sites. **Fig.S5.** Annotation of the P10 site with CRISPR/Cas9 test features and DNA-Seq validation of genome-editing outcome in the P10-KO stable pool. **Fig.S6.** Annotation of the P11 site with CRISPR/Cas9 test features and DNA-Seq validation of genome-editing outcome in the P11-KO stable pool. **Fig.S7.** Annotation of the P4 site with CRISPR/Cas9 test features and DNA-Seq validation of genome-editing outcome in the P4-KO stable pool. **Fig.S8.** Alignment H3K27ac occupancy in the 8q24-MYC gene locus of typical PCa cells. **Fig.S9.** Annotate 8q24-MYC gene locus with prostate cancer risk SNP. **Fig.S10.** Re-annotate Fig.6A datasets based on the Hg38 reference. **Fig.S11.** Bioinformatics analyses of global androgen effects on distribution of AR, H3K27ac, FoxA1 and HoxB13 in VCaP cells. **Fig.S12.** FoxA1 and HoxB13 manipulation in AR(+) PCa cells impacted 8q24-MYC gene locus activities and MYC gene expression. **Additional information on materials and methods.**


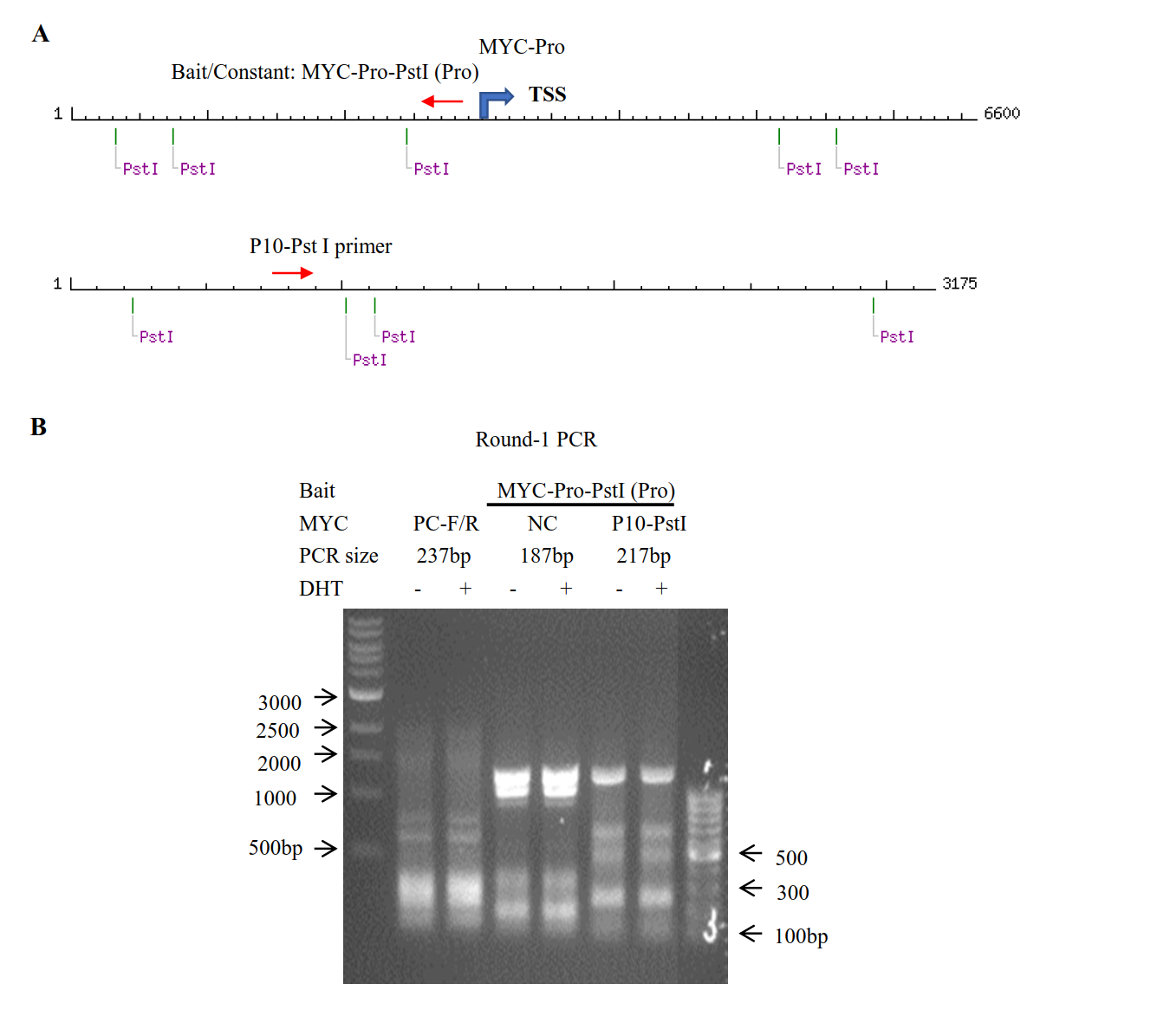


**Fig.S1. 3C analyses of the chromosomal interactions between MYC promoter and P10 in VCaP cells. A,** Schematic showing of the 3C assay strategy based on PstI digestion, aiming to address the interaction between MYC promoter (MYC-Pro) and the ABS P10. The proximal PstI sites (CTGCAG) and the bait and target primers in 3C PCR analyses are also shown. TSS: transcription starting site. **B,** nest-PCR analysis of 3C signals, and this is the round-1 PCR results. PC-F/R: positive loading control primers that target MYC-Pro locus without PstI site in the amplicon. NC: negative control primer that target a distal Chr8 site with proximal PstI site but no AR binding site.


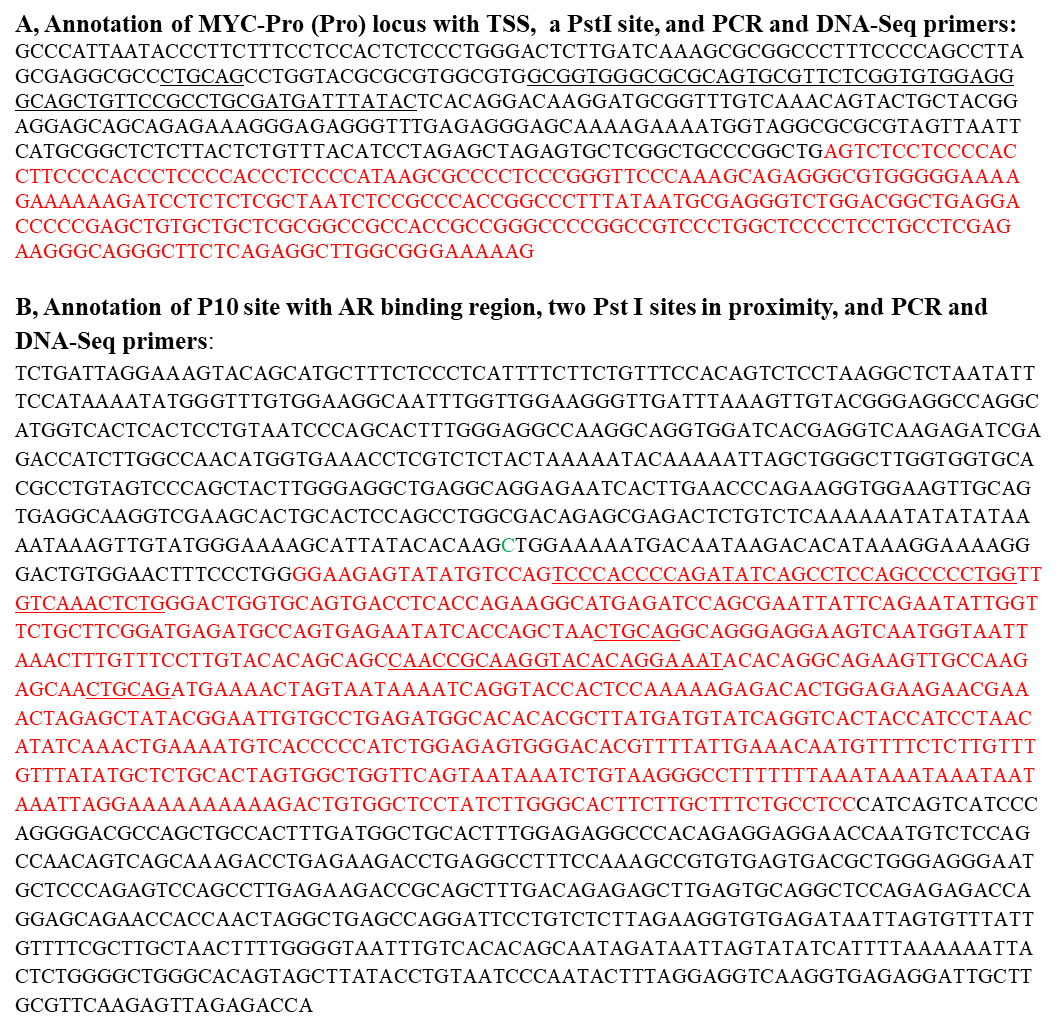


**Fig.S2. Annotation of MYC-Pro and P10 sites with 3C assay features. A,** Alignment of MYC-Pro locus with MYC TSS and transcripts (in red), a proximal upstream PstI site (CTGCAG, underlined), and PCR, nest-PCR, and ddPCR primers (underlined) that was examined in the 3C studies. **B,** Alignment of P10 with AR binding peak region (in red) and two 3C PstI sites in proximity (underlined). Also aligned are PCR and DNA-Seq primers (underlined). Based on these two PstI sites, two theoretical major 3C PCR products of MYC-Pro-P10 hybrids were confirmed by DNA electrophoresis (Figure 3D) and the DNA-Seq annotation results (See also Figure 3 and Additional Figures Fig.S1 and S3 for more information).


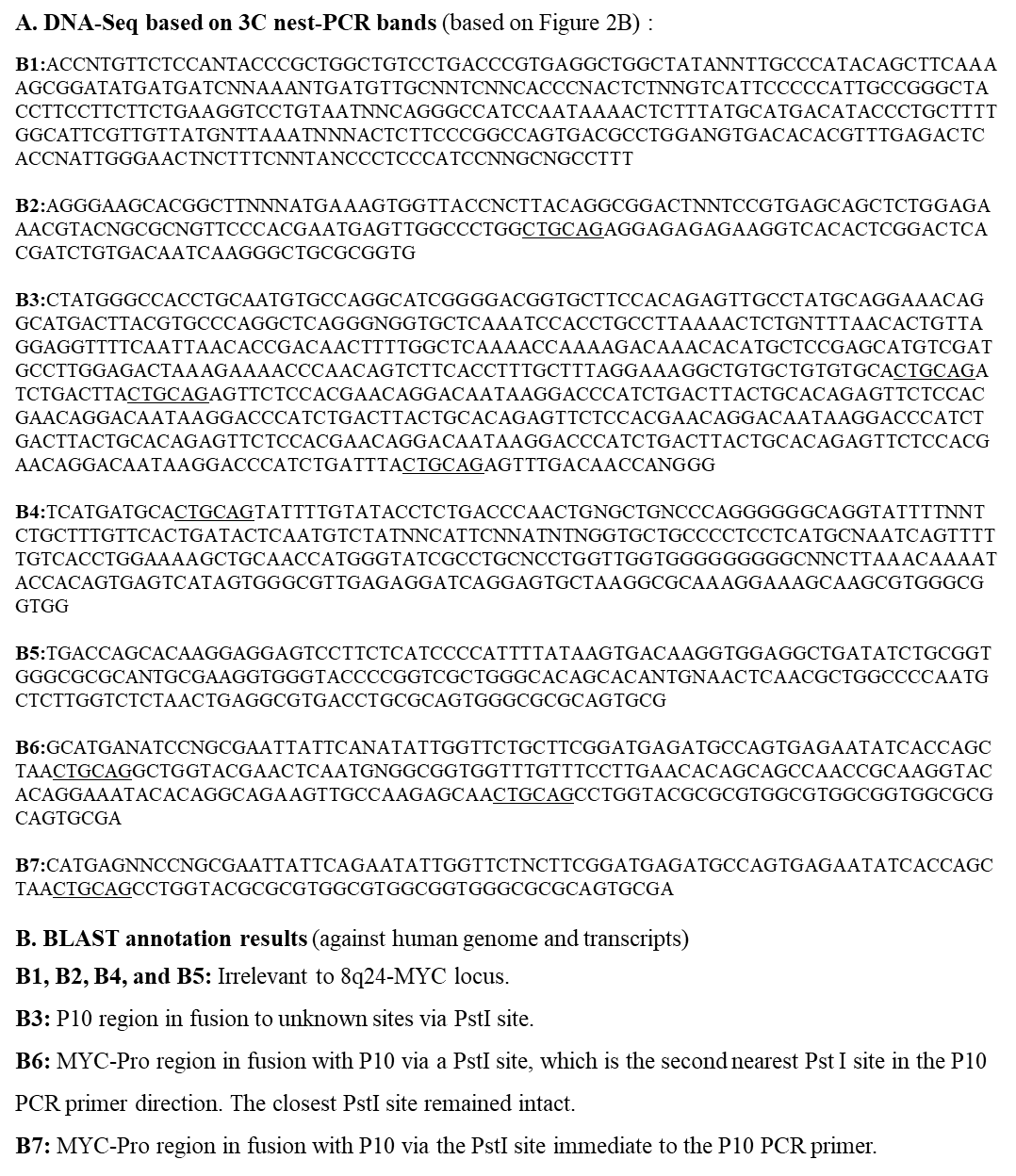


**Fig.S3. DNA-Seq validation of DHT-induced MYC-Pro-P10 looping. A,** DNA-Seq read-outs with the PstI site (CTGCAG) underlined. **B,** DNA-Seq annotation based on BLAST against human genome and transcripts. The overall conclusion: B6 and B7 are both confirmed as MYC-Pro-P10 hybrids fused at two distinct P10-proximal PstI sites, respectively. These findings, together with the ddPCR and SYBR RT-qPCR results, firmly established androgen-motivated spatial interaction between MYC-Pro and P10. (See also Figure 3 and Additional Figures Fig.S1-S2 for more information).


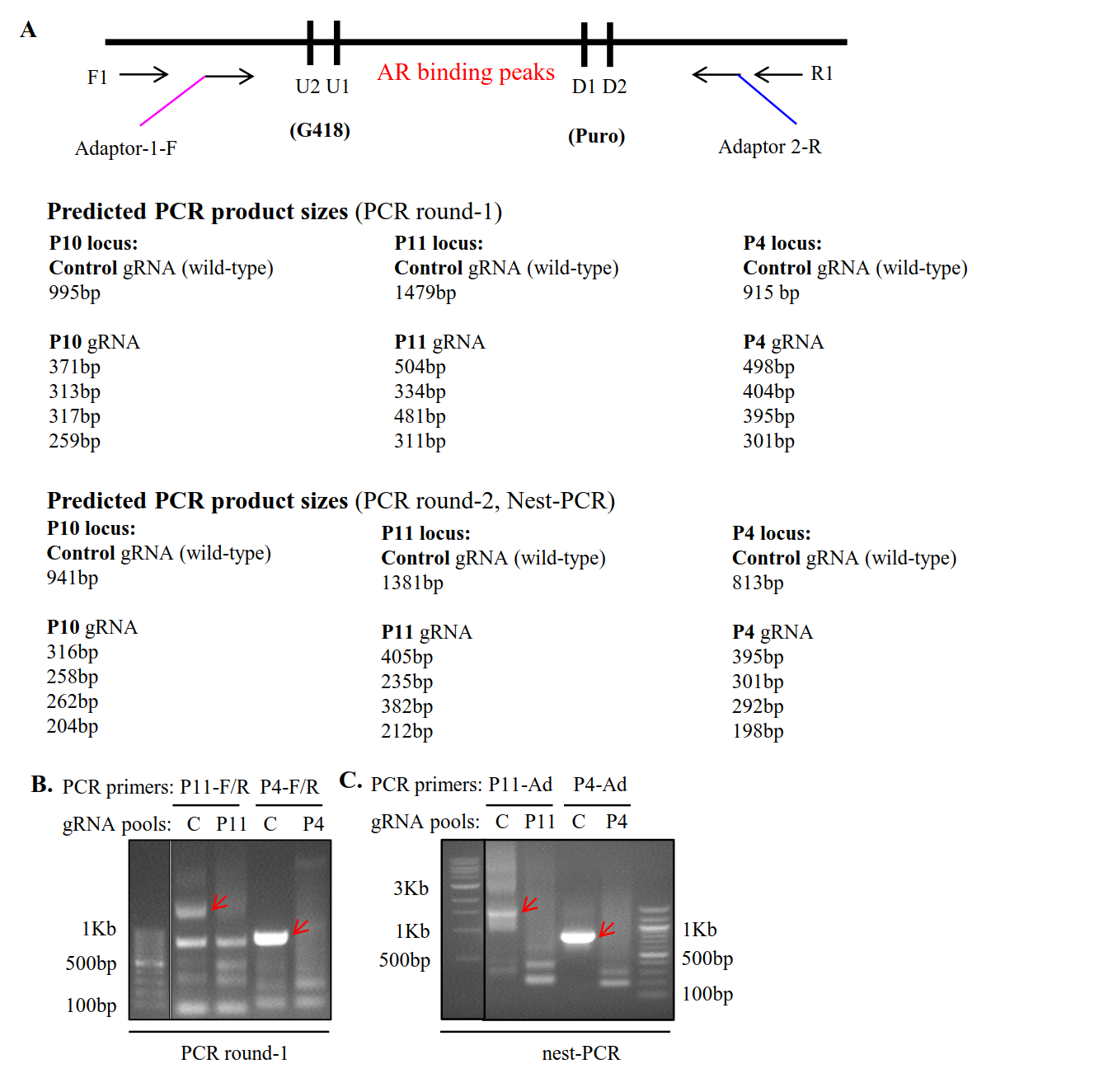


**Fig.S4**. **PCR and DNA-Seq validation of CRISPR/Cas9-mediated genomic KO of P10, P11 and P4 sites. A,** Schematic drawing of KO strategy, nest-PCR primers, and theoretical PCR product sizes based on Cas9 cleavage sites in stable pools. The gene editing and PCR outcomes depend on the cleavage efficacy at U1/U2 site and D1/D2 sites, respectively. **B-C,** two rounds PCR gel imaging results based on control (C) versus P11 and P4 stable KO pools. Arrow heads indicate the wild-type PCR bands. Ad: adaptor. For more information see Fig.4-5 and Additional Figures Figure S5–S7.


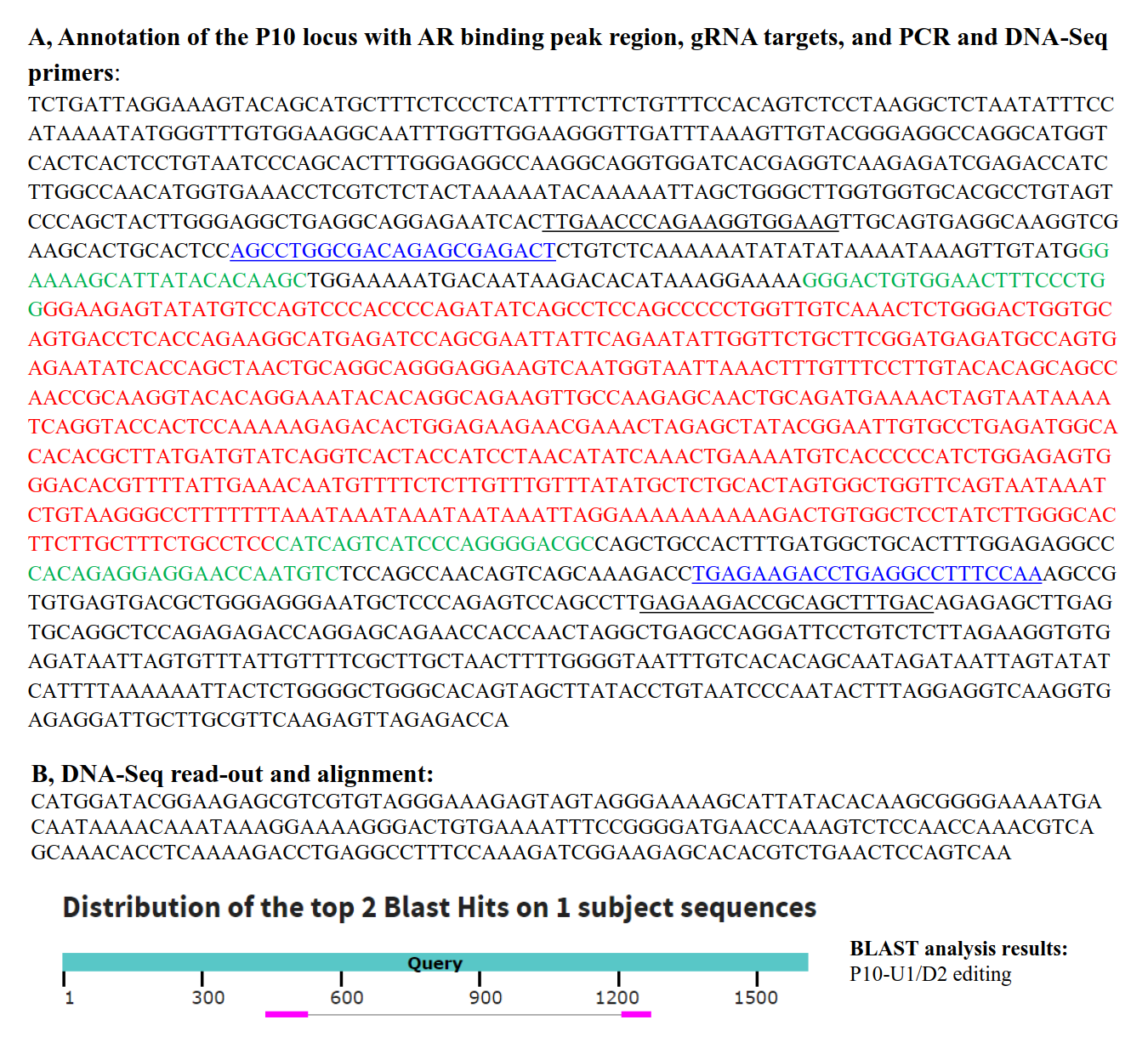


**Fig.S5. Annotation of the P10 site with CRISPR/Cas9 test features and DNA-Seq validation of genome-editing outcome in the P10-KO stable pool. A,** The AR binding peak region is in red. Also aligned are gRNA targets (in green) and PCR and DNA-Seq primers (underlined). Colored are PCR (F1/R1 in black and adaptor-F/R in blue), DNA-Seq primers (adaptor-F/R, in blue) and gRNA oligos (U2/U1, D1/D2, in green). The theoretical positions of gRNA cleavage sites are: 464 (U2 gRNA), 518 (U1 gRNA), 1141 (D1 gRNA), and 1199 (D2 gRNA), respectively. **B,** Blast annotation of one verification DNA-Seq read-out indicated the editing mainly occurred at theoretical positions of U1/D2 gRNA cleavage sites. (See also Figure 4 and Additional Figure Fig.S4 for more information).


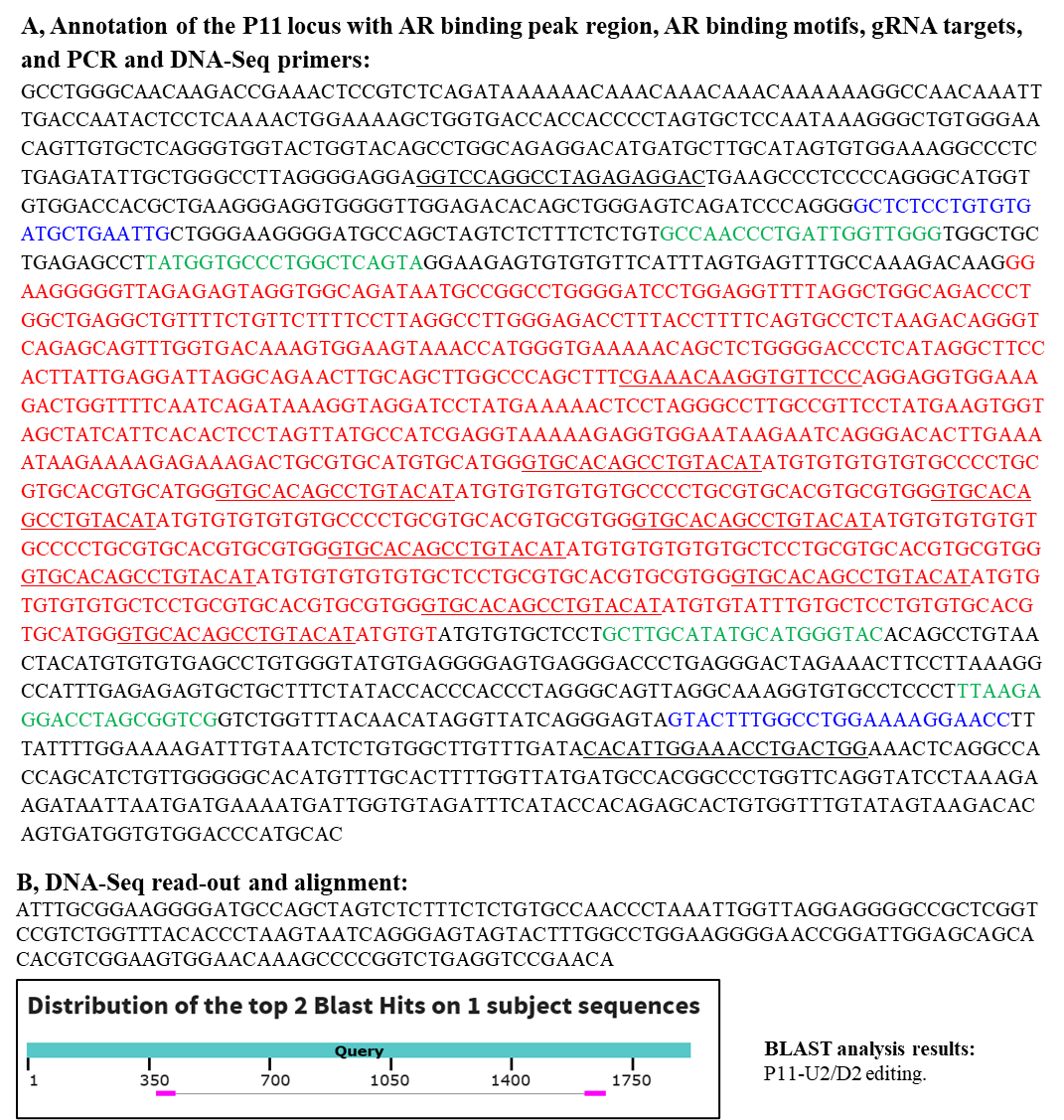


**Fig.S6. Annotation of the P11 site with CRISPR/Cas9 test features and DNA-Seq validation of genome-editing outcome in the P11-KO stable pool. A,** The AR binding peak region is in red. Underlined are PCR (F1/R1, in black and adaptor-F/R, in blue) and DNA-Seq primers (adaptor-F/R, in blue). Also colored are gRNA targets (U2/U1, D1/D2, in green). The theoretical positions of gRNA cleavage sites are: 420 (U2 gRNA), 442 (U1 gRNA), 1417 (D1 gRNA), and 1587 (D2 gRNA), respectively. Significantly, the P11 central AR peak contains at least 10 potential AR binding motifs that are highly repetitive, as underlined in the red area. **B,** Blast annotation of DNA-Seq indicated the editing mainly occurred at theoretical positions of U2/D2 gRNA cleavage sites. (See also Figure 5 and Additional Figure Fig.S4 for more information).


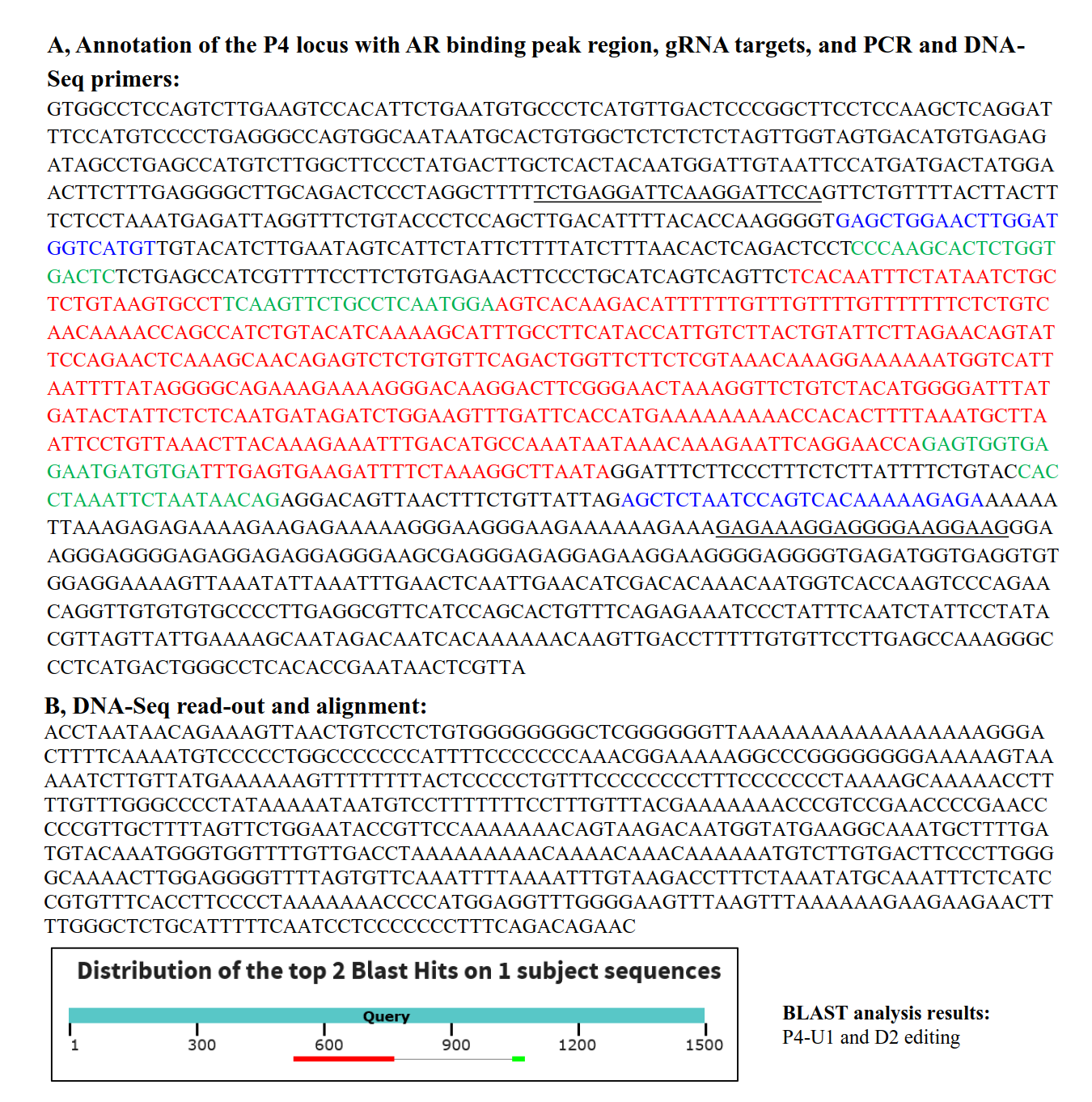


**Fig.S7. Annotation of the P4 site with CRISPR/Cas9 test features and DNA-Seq validation of genome-editing outcome in the P4-KO stable pool. A,** The AR binding peak region is in red. Also aligned are gRNA and PCR and DNA-Seq primers. Underlined are PCR (F1/R1, in black and adaptor-F/R, in blue) and DNA-Seq primers (adaptor-F/R, in blue). Also colored are gRNA targets (U2/U1, D1/D2, in green). The theoretical positions of gRNA cleavage sites are: 434 (U2 gRNA), 537 (U1 gRNA), 954 (D1 gRNA), and 1048 (D2 gRNA), respectively. **B,** Blast annotation of DNA-Seq indicated the editing mainly occurred at theoretical positions of U1/D2 gRNA cleavage sites. See also Figure 5 and Additional Figure Fig.S4 for more information.

**Fig.S8. Alignment H3K27ac occupancy in the 8q24-MYC gene locus of typical PCa cells.** The H3K27ac ChIP-Seq datasets (GSE171589 and GSE143653) of two AR(+) (LNCaP and VCaP) and two AR(-) (PC3 and DU145) cell lines were annotated based on the human genome build 19 (Hg19) by Bowtie2 with default parameters. ChIP-seq reads were first trimmed by Trim Galore and then aligned to Hg19. The bam files were then subjected to MACS2 for peak calling with the parameter “–SPMR” on and “–keep-dup = 1”. Significant peaks were identified by q-value < 0.05. UCSC bedGraphToBigWig tool was used to convert resultant bedgraph files to bigWig files, which were then used for IGV peak visualization. As shown, the left arm of the 8q24-MYC gene locus has enhancer enrichment in AR(+) (LNCaP and VCaP) but not AR(-) (PC3 and DU145) cell lines.


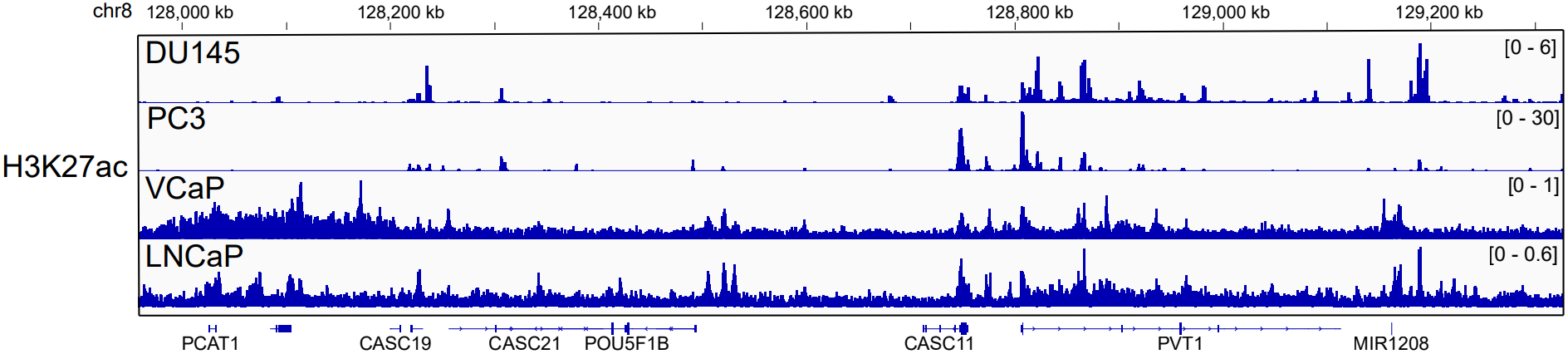


**Fig.S9. Annotate 8q24-MYC gene locus with prostate cancer risk SNP.** The VCaP ChIP-seq datasets were annotated based on the human genome build 19 (Hg19) by Bowtie2 with default parameters for IGV peak visualization. PCa risk SNP alignment was based on the GWAS Catalog database. As shown, left arm of the 8q24-MYC gene locus is enriched in PCa-risk SNP and ABS-associated enhancers (including the PCAT1 SE).


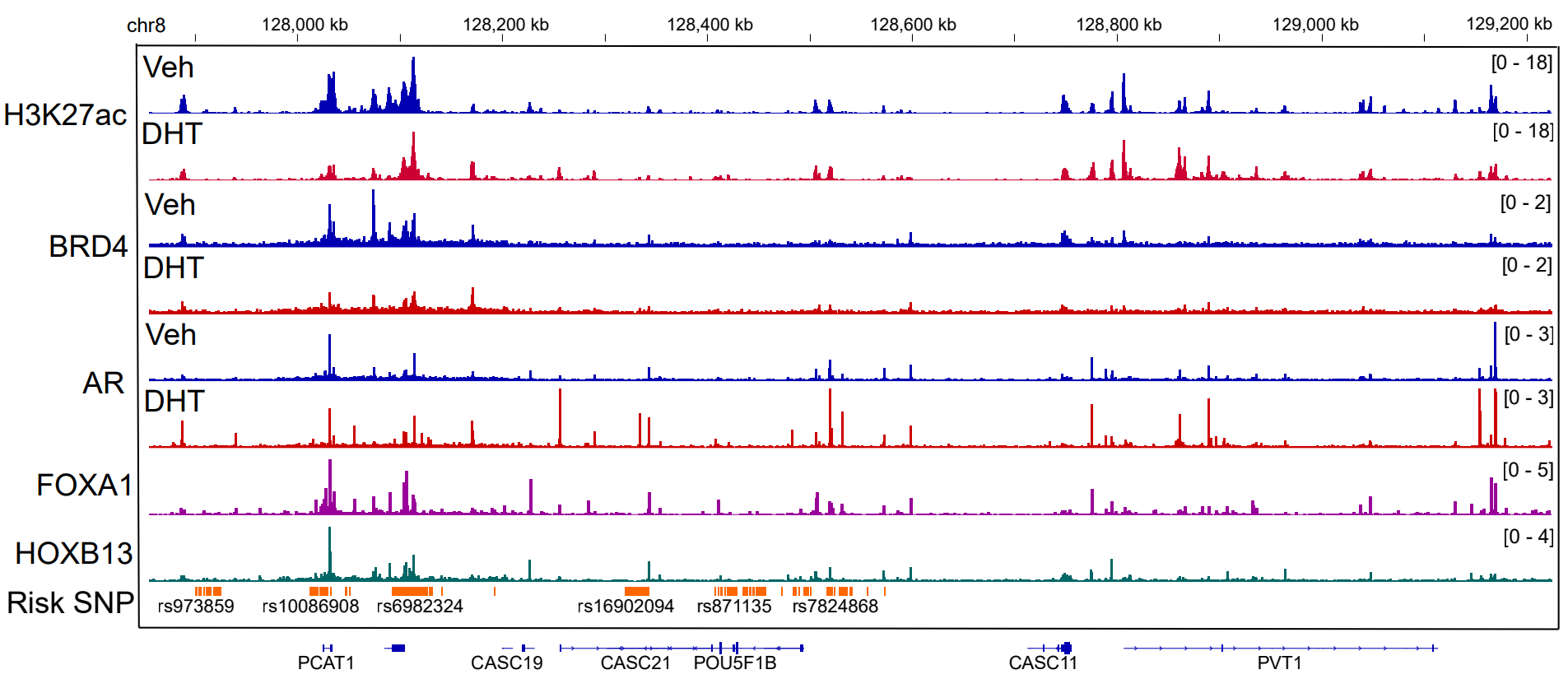


**Fig.S10.** **Re-annotate Fig.6A datasets based on the Hg38 reference.** To verify the reproducibility of data interpretation and annotation, the same VCaP ChIP-seq datasets of Fig.6A (based on the Hg19 reference) was subjected to re-alignment. Accordingly, ChIP-seq reads were first trimmed by Trim Galore and then aligned to the human genome build 38 (Hg38) by Bowtie2 with default parameters. The bam files were then subjected to MACS2 for peak calling with the parameter “–SPMR” on and “–keep-dup = 1”. Significant peaks were identified by q-value < 0.05. UCSC bedGraphToBigWig tool was used to convert resultant bedgraph files to bigWig files, which were then used for IGV peak visualization. As shown, alignments based on Hg19 and Hg38 references obtained consistent co-factor distribution profiles, validating the reliability of our analysis.


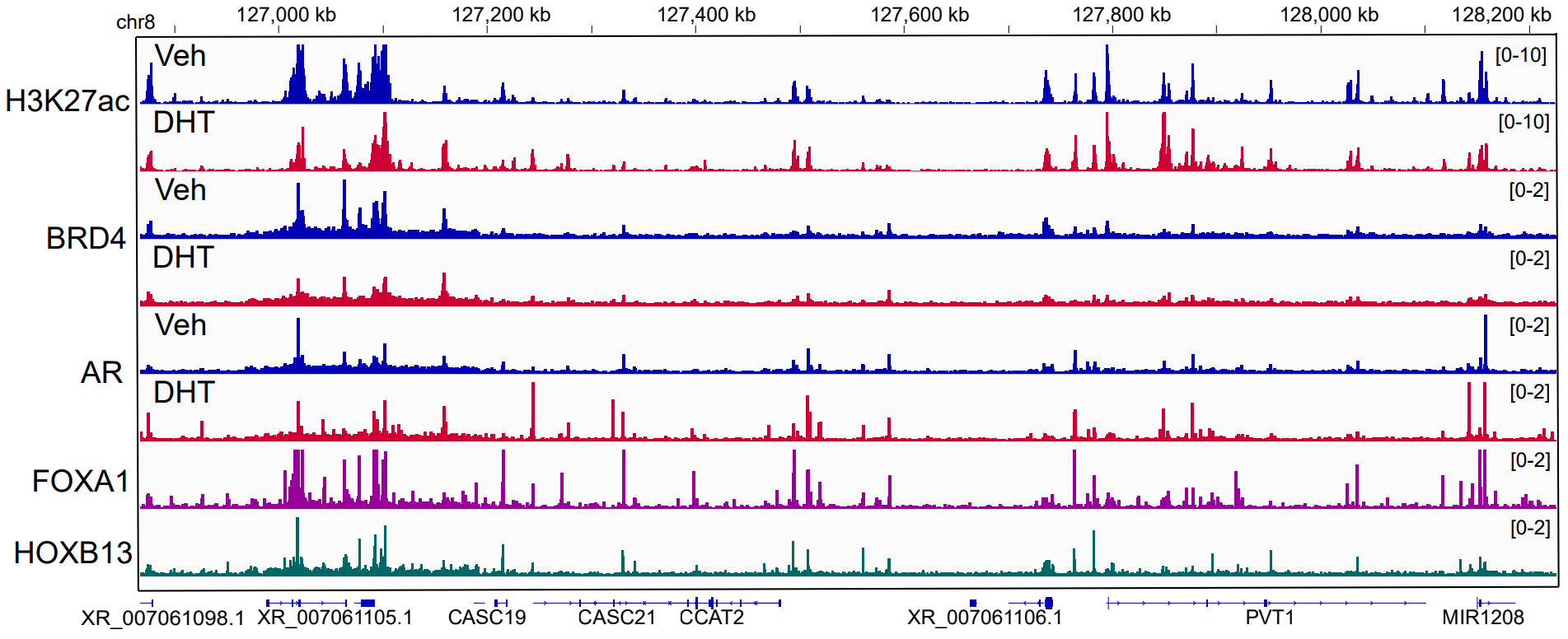


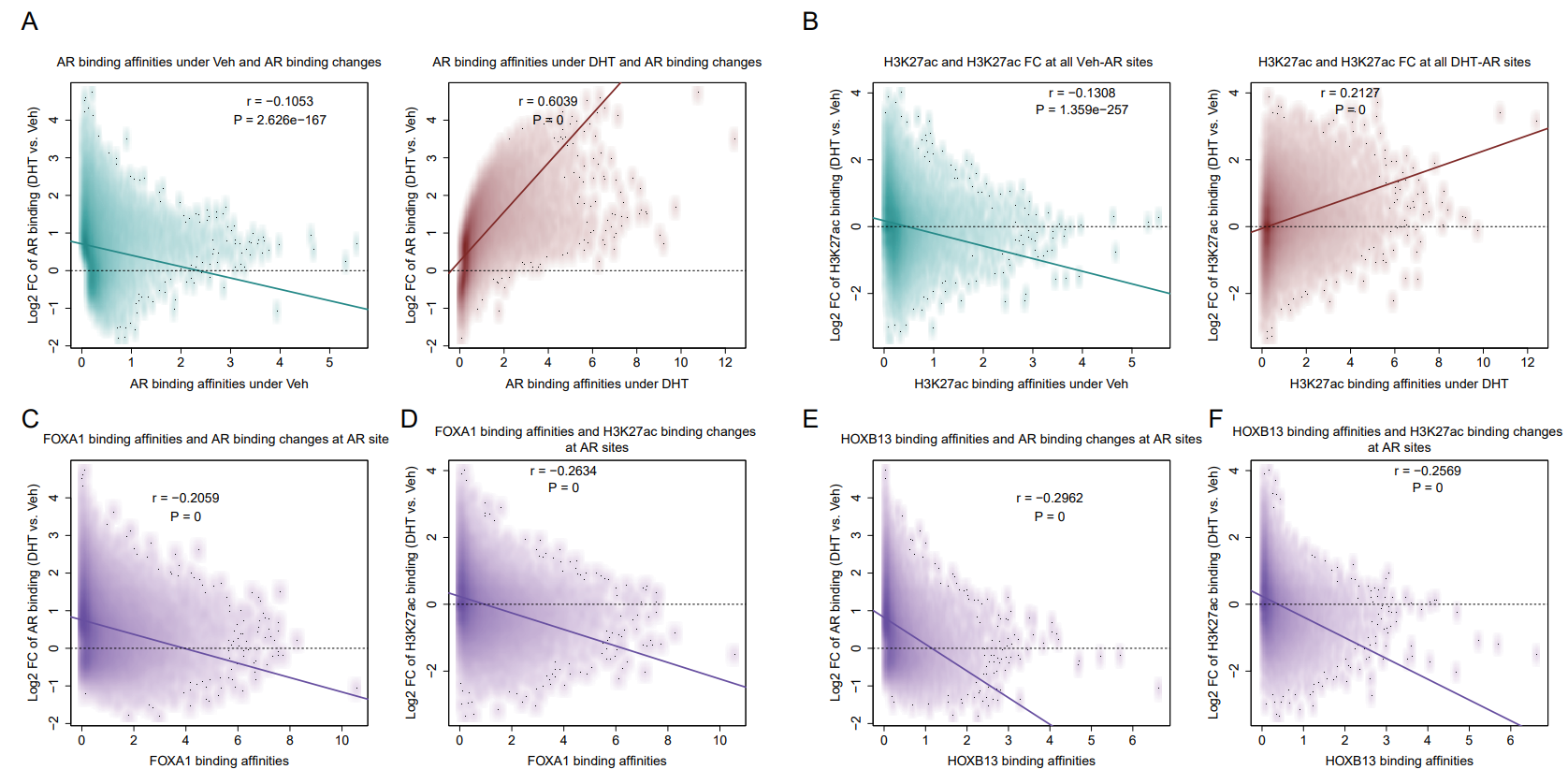


**Fig.S11. Bioinformatics analyses of global androgen effects on distribution of AR, H3K27ac, FoxA1 and HoxB13 in VCaP cells. A**, The correlation between AR binding affinities and AR binding fold changes (FC: DHT vs. Veh) at all Veh-AR sites and all DHT-AR sites, respectively. **B**, The correlation between H3K27ac signal and H3K27ac signal fold changes (DHT vs. Veh) at all Veh-AR sites and all DHT-AR sites, respectively. **C**, The correlation between FOXA1 binding affinities and AR binding fold changes (DHT vs. Veh) at all AR sites (combination of both Veh and DHT AR sites). **D**, The correlation between FOXA1 binding affinities and H3K27ac signal fold changes (DHT vs. Veh) at all AR sites. **E**, The correlation between HOXB13 binding affinities and AR binding fold changes (DHT vs. Veh) at all AR sites. **F**, The correlation between HOXB13 binding affinities and H3K27ac signal fold changes (DHT vs. Veh) at all AR sites.

**Fig.S12. FoxA1 and HoxB13 manipulation in AR(+) PCa cells impacted 8q24-MYC gene locus activities and MYC gene expression. A-B)** LNCaP-abl cell line was subjected to FoxA1 siRNA KO, followed by H3K27ac ChIP-Seq and RNA-Seq assays (GSE72467 and GSE72534). **C-D)** LNCaP stable lines were generated with tetracycline-inducible expression of V5-tagged FKHD-MSs or WT FOXA1, followed by ChIP-Seq and RNA-Seq assays (GSE133384 and GSE133387). **E-F)** LNCaP was subjected to HoxB13 siRNA KD and ectopic expression of HoxB13 WT versus G84E mutant (two repeats for each sample set), followed by ChIP-Seq and RNA-Seq assays (GSE153583 and GSE153585). ChIP-Seq alignment was based on the Hg19 reference for IGV peak visualization. For RNA-Seq analysis, raw sequencing reads were first trimmed by Trim Galore and clean reads were next aligned using STAR with parameters “–outSAMattributes NH HI NM MD–outSAMstrandField intronMotif–quantMode GeneCounts”. Fragments Per Kilobase of exon model per Million mapped fragments (FPKM) were then calculated based on gene read count.

**Additional information on materials and methods.**

**PCR and RT-PCR kits, DNA purification kits and DNA rulers:** 1, PCR and nest-PCR kit: Phusion Hot Start Flex DNA Pol (NEB, 0535); 2, RT-qPCR (DNA template) kit: Power SYBR Green PCR Master Mix (Thermo Fisher, Cat. 4367659); 3, DNA purification kit: QIAquick PCR Purification Kit (Cat. No. 28106, Qiagen; Germantown, MD, USA) and Gel purification kit (Qiagen, Cat. No. 28506). 4, 1Kb DNA ladder (New England Biolabs, N3232S); 100 bp DNA Ladder (Thermo Scientific, SM0243).

**PCR and RT-PCR parameters and DNA-Seq:** The PCR analysis was performed with the Step-One-Plus Real-time PCR system (Applied Biosystems). PCR amplification using Phusion kit (NEB, Catalog 0535) and the thermocycling conditions for PCR round-1 is (1x, 98C, 2min; 40x (98C, 15sec; 63C, 30sec; 72C, 30sec); 1x, 72C, 7min; and hold at 4C) and for nest-PCR is (1x, 98C, 30sec; 40x (98C, 15sec; 63C, 30sec; 72C, 20sec); 1x, 72C, 7min; and hold at 4C). Both runs start with a reaction volume of 50ul and 1.5ul of round-1 PCR products was used as the template for nest-PCR. Nest-PCR products (with regular primers) were purified for regular Sanger sequencing; nest-PCR products (with primers containing adaptors) were purified and submitted for sequencing with the Genewiz Amplicon EZ program.

**PCR and RT-PCR primers and probes, and gRNA construct oligos:** The uniqueness of all primers and oligos in this study has been validated by BLAST against human genome and transcripts.

**1, ChIP-qPCR assay primers:**

Myc_pro_F, AGGGCTTCTCAGAGGCTTG

Myc_pro_R, CGGCTCTTCCACCCTAGC

P10-F, CCGCAAGGTACACAGGAAAT

P10-R, CCAGTGTCTCTTTTTGGAGTGG

PCAT1-Pro-F, CTCCGAACCACACATGGATA

PCAT1-Pro-R, CCTTGCCACTTTCCTTTCCT

PCAT1-ABS1-F (P1), GGTCAGGGATGTTGATTTAGC

PCAT1-ABS1-R (P1), AAAGTTTGGGTTTATTGCAAATG

CCAT1-ABS1-F (P4), TCCAGAACTCAAAGCAACAGAG

CCAT1-ABS1-R (P4), CCCTTTTCTTTCTGCCCCTA

CASC8-ABS1-F (P7), AATCCAGCACTGCTTGTGG

CASC8-ABS1-R (P7), CAAATGAGCATGACTGTGTCC

PVT1-Pro-F, GCATGGAGCTTCGTTCAAGT

PVT1-Pro-R, TGTCATTCCAGTGCATGGTT

PVT1-ABS1-F (P11), CCAGCTTTCGAAACAAGGTG

PVT1-ABS1-R (P11), GCAAGGCCCTAGGAGTTTTT

PVT1-ABS2-F (P13), AACAGCCCACAGCATTTTTC

PVT1-ABS2-R (P13), TCACAGTGTTCTGGGTCAGC

PVT1-ABS3-F (P14), TCTCAGTGCTGTGTGCTGTTT

PVT1-ABS3-R (P14), CCTGTTTCTCAGCAGCAACT

PSA-Enh-F, GCCTGGATCTGAGAGAGATATCATC

PSA-Enh-R, ACACCTTTTTTTTTCTGGATTGTTG

NC-F, TCCTGCATTCCATAGCTTTT (Non-specific genomic control site on Chr1)

NC-R, TCTGATCACAATGGAATGAAACT (Non-specific genomic control site on Chr1)

**2, PCR primers for 3C assay** (round-1 PCR)**:**

3C-MYC-Pro-PstI: AACAGCTGCCCTCCACACCGAGAA

3C-MYC-P10-PstI: TCCCACCCCAGATATCAGCCTCCA

3C-MYC-Pro-PC-F, TTCTCGGTGTGGAGGGCAGCTGTT

3C-MYC-Pro-PC-R, GGTGGGGAAGGTGGGGAGGAGACT

3C-MYC-Pro-NC: AGAGCTCAGCCAAGCTGGCACGAA

**3, PCR primers for 3C assay** (round-2 PCR, Nest-PCR)**:**

Nest-MYC-Pro-PstI, CGCACTGCGCGCCCACCGC

Nest-MYC-P10-PstI, GCCCCCTGGTTGTCAAACTCTG

Nest-MYC-Pro-PC-F, CCGCCTGCGATGATTTATACTCAC

Nest-MYC-Pro-PC-R, CAGCCGGGCAGCCGAGCACT

Nest-MYC-Pro-NC, GTGGTATTCCCATCATACTCTCTC

**4, ddPCR and RT-qPCR primers/probes for 3C assay:**

Droplet digital PCR (ddPCR): The ddPCR was performed on the Bio-Rad QX200 AutoDG Droplet Digital PCR System (Bio-Rad, Hercules, CA, USA) that uses a C1000 Touch Thermal Cycler with 96–Deep Well Reaction Module. The ddPCR Probe no UNG cycling conditions: 95 °C/10 min; 40 cycles of 94 °C/30 s and 60 °C/1 min; 98 °C/10 min. The MYC-promoter was used as bait (constant) that was paired with primer specifically located in target regions. Specific probe signal was normalized to that of the copy number reference RPPH1 that does not contain a PstI site in the amplicon.

q3C-MYC-pro-PstI-C, GTATAAATCATCGCAGGCGGAAC

FAM-MYC-Pst-QSY, FAM-TGCAGCCTGGTACGCGCGTGGCGT-QSY

q3C-P10-PstI, CAACCGCAAGGTACACAGGAAAT

Copy number reference is RPPH1 (RNase P).

q3C-hGAPDH-F, CCCTTTCACCATTAGGGACCTTC

q3C-hGAPDH-R, GCTAAGTTTAGCCTGCCTGGTGA

**5, gRNA expression constructs and** **oligos:**

Lenti-gRNA-Neo vectors (LGN, Addgene, Catalog. 104992) were constructed to express a pair of upstream sgRNAs (U1/U2); Lenti-gRNA-Puro vectors (LGP, Addgene, Catalog. 104990) were generated to express a pair of downstream sgRNAs (D1/D2), respectively. The cloning is based on vector BsmBI digestion (NEB, R0580L), oligo annealing and ligation with T4 DNA ligase (NEB, M0202).

**Control gRNA site (non-specific)** **oligos:**

gRNA-C1-F: CACCGGTAGCGAACGTGTCCGGCGT

gRNA-C1-R: AAACACGCCGGACACGTTCGCTACC

gRNA-C2-F: CACCGGACCGGAACGATCTCGCGTA

gRNA-C2-R: AAACTACGCGAGATCGTTCCGGTCC

**P10 site oligos:**

P10-gRNA-U1-F: CACCGGGACTGTGGAACTTTCCCTG

P10-gRNA-U1-R: AAACCAGGGAAAGTTCCACAGTCCC

P10-gRNA-U2-F: CACCGGAAAAGCATTATACACAAGC

P10-gRNA-U2-R: AAACGCTTGTGTATAATGCTTTTCC

P10-gRNA-D1-F: CACCGCGTCCCCTGGGATGACTGA

P10-gRNA-D1-R: AAACATCAGTCATCCCAGGGGACGC

P10-gRNA-D2-F: CACCGGACATTGGTTCCTCCTCTGT

P10-gRNA-D2-R: AAACACAGAGGAGGAACCAATGTCC

**P11 site oligos:**

P11-gRNA-U1-F: CACCGTACTGAGCCAGGGCACCATA

P11-gRNA-U1-R: AAACTATGGTGCCCTGGCTCAGTAC

P11-gRNA-U2-F: CACCGGCCAACCCTGATTGGTTGGG

P11-gRNA-U2-R: AAACCCCAACCAATCAGGGTTGGCC

P11-gRNA-D1-F: CACCGGTACCCATGCATATGCAAGC

P11-gRNA-D1-R: AAACGCTTGCATATGCATGGGTACC

P11-gRNA-D2-F: CACCGCGACCGCTAGGTCCTCTTAA

P11-gRNA-D2-R: AAACTTAAGAGGACCTAGCGGTCGC

**P4 site oligos:**

P4-gRNA-U1-F: CACCGTCCATTGAGGCAGAACTTGA

P4-gRNA-U1-R: AAACTCAAGTTCTGCCTCAATGGAC

P4-gRNA-U2-F: CACCGGAGTCACCAGAGTGCTTGGG

P4-gRNA-U2-R: AAACCCCAAGCACTCTGGTGACTCC

P4-gRNA-D1-F: CACCGTCACATCATTCTCACCACTC

P4-gRNA-D1-R: AAACGAGTGGTGAGAATGATGTGAC

P4-gRNA-D2-F: CACCGCACCTAAATTCTAATAACAG

P4-gRNA-D2-R: AAACCTGTTATTAGAATTTAGGTGC

**P11-M site oligos** (targeting multiple AR peaks):

P11-M-gRNA-U1-F, CACCGTTCTCAAGTCCCTATAGAAG

P11-M-gRNA-U1-R, AAACCTTCTATAGGGACTTGAGAAC

P11-M-gRNA-U2-F, CACCGAGGAGTCATGCCCAAGATAC

P11-M-gRNA-U2-R, AAACGTATCTTGGGCATGACTCCTC

P11-M-gRNA-D1-F, CACCGCTCACAAAATACGAAGACGC

P11-M-gRNA-D1-R, AAACGCGTCTTCGTATTTTGTGAGC

P11-M-gRNA-D2-F, CACCGCTGGGTCGCGTGTCCTGGCA

P11-M-gRNA-D2-R, AAACTGCCAGGACACGCGACCCAGC

**P1 site oligos:**

P1-gRNA-U1-F, CACCGCTAGAATGCGGTCCAAGCAT

P1-gRNA-U1-R, AAACATGCTTGGACCGCATTCTAGC

P1-gRNA-U2-F, CACCGTCGTTAGAGAAGCCAATGCT

P1-gRNA-U2-R, AAACAGCATTGGCTTCTCTAACGAC

P1-gRNA-D1-F, CACCGTTTCCAGCTTCTGGTCTGAT

P1-gRNA-D1-R, AAACATCAGACCAGAAGCTGGAAAC

P1-gRNA-D2-F, CACCGCAGGGGATAAATATCCTAGC

P1-gRNA-D2-R, AAACGCTAGGATATTTATCCCCTGC

**CRE2 site oligos:**

CRE2-gRNA-U1-F, CACCGCATGGTGAAGGTGGCTAAAG

CRE2-gRNA-U1-R, AAACCTTTAGCCACCTTCACCATGC

CRE2-gRNA-U2-F, CACCGGTAATAGATGGCCTAATATT

CRE2-gRNA-U2-R, AAACAATATTAGGCCATCTATTACC

CRE2-gRNA-D1-F, CACCGATAAGGCAGAGGAAACCTTT

CRE2-gRNA-D1-R, AAACAAAGGTTTCCTCTGCCTTATC

CRE2-gRNA-D2-F, CACCGGAGTTGCTAAATTTGCTAAG

CRE2-gRNA-D2-R, AAACCTTAGCAAATTTAGCAACTCC

**P2 site oligos:**

P2-gRNA-U1-F, CACCGCTCATGCTGTATTTCTATTA

P2-gRNA-U1-R, AAACTAATAGAAATACAGCATGAGC

P2-gRNA-U2-F, CACCGTGCTAGTCACTACCTTAAAA

P2-gRNA-U2-R, AAACTTTTAAGGTAGTGACTAGCAC

P2-gRNA-D1-F, CACCGTATACATATGATCAATGGAG

P2-gRNA-D1-R, AAACCTCCATTGATCATATGTATAC

P2-gRNA-D2-F, CACCGATATGTCATAGTTGATTCTC

P2-gRNA-D2-R, AAACGAGAATCAACTATGACATATC

**6, Primers for PCR and DNA-Seq validation of CRISPR/Cas9-based genomic KO:**

**P10 site:**

Primers for P10 site (round-1 PCR):

P10-F1: TTGAACCCAGAAGGTGGAAG

P10-R1: GTCAAAGCTGCGGTCTTCTC

Adaptor-primers (for P10 site Nest-PCR and DNA-Seq) (Adaptor part is underlined):

Adaptor-P10-F (Ad-P10-F):

ACACTCTTTCCCTACACGACGCTCTTCCGATCTAGCCTGGCGACAGAGCGAGACT

Adaptor-P10-R (Ad-P10-R):

GACTGGAGTTCAGACGTGTGCTCTTCCGATCTTTGGAAAGGCCTCAGGTCTTCTCA

**P11 site:**

Primers for P11 site (round-1 PCR):

P11-F1: GGTCCAGGCCTAGAGAGGAC

P11-R1: CCAGTCAGGTTTCCAATGTG

Adaptor primers (for P11 site Nest-PCR and DNA-Seq) (Adaptor part is underlined):

Adaptor-P11-F (Ad-P11-F):

ACACTCTTTCCCTACACGACGCTCTTCCGATCTGCTCTCCTGTGTGATGCTGAATTG

Adaptor-P11-R (Ad-P11-R):

GACTGGAGTTCAGACGTGTGCTCTTCCGATCTGGTTCCTTTTCCAGGCCAAAGTAC

**P4 site:**

Primers for P4 site (round-1 PCR):

P4-F1: TCTGAGGATTCAAGGATTCCA

P4-R1: CTTCCTTCCCCTCCTTTCTC

Adaptor primers (for P4 site Nest-PCR and DNA-Seq) (Adaptor part is underlined):

Adaptor-P4-F (Ad-P4-F):

ACACTCTTTCCCTACACGACGCTCTTCCGATCTGAGCTGGAACTTGGATGGTCATGT

Adaptor-P4-R (Ad-P4-R):

GACTGGAGTTCAGACGTGTGCTCTTCCGATCTTCTCTTTTTGTGACTGGATTAGAGCT

**Reference control:**

MYC-Ctr-F: TTCTCGGTGTGGAGGGCAGCTGTT

MYC-Ctr-R: GGTGGGGAAGGTGGGGAGGAGACT

PSA-Ctr-F: TGTCCTGGAGAGGGTGGAGGTGGA

PSA-Ctr-R: AGGTGGGCACTGAGACTGCACTGG
